# Supplementary material for: Microtubule nucleation and γTuRC centrosome localization in interphase cells require ch-TOG
Source: Nat Commun. 2023 Jan 26;14:289. doi: 10.1038/s41467-023-35955-w (PMC9879976; doi:10.1038/s41467-023-35955-w)
Supplement: Supplementary file 1 — Supplementary Information [file 41467_2023_35955_MOESM1_ESM.pdf]

Supplementary Information

Microtubule nucleation and γTuRC centrosome localization in interphase cells require ch-TOG

Aamir Ali[1], Chithran Vineethakumari[1], Cristina Lacasa[1] and Jens Lüders[1,*]

Affiliation:

[1] Institute for Research in Biomedicine (IRB Barcelona), The Barcelona Institute of Science and Technology, Barcelona 08028, Spain.

* correspondence: jens.luders@irbbarcelona.org

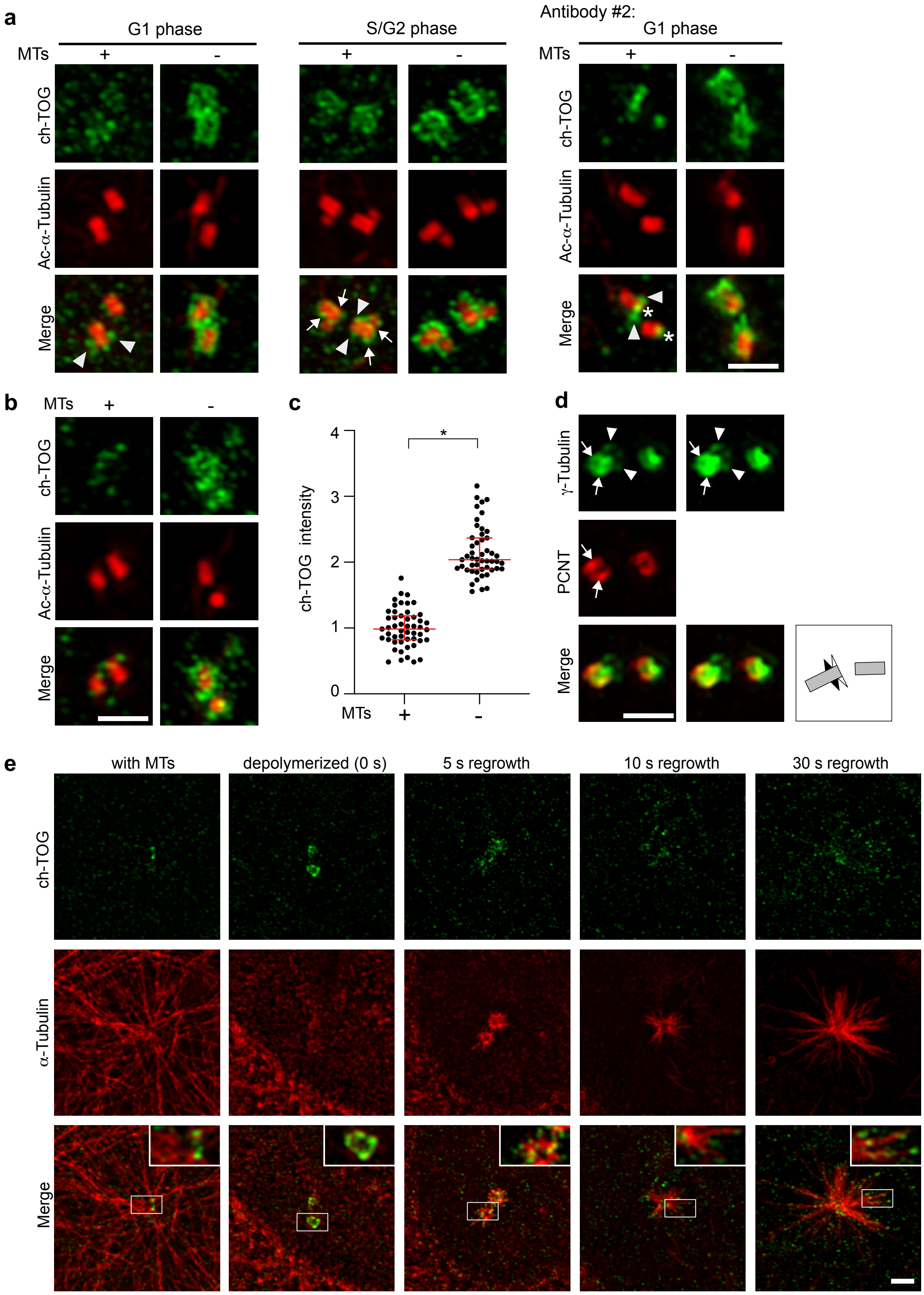

Supplementary Fig. 1. CKPA5 transiently localizes to the PCM in different cell lines and cell-cycle stages. a U2OS cells with and without microtubules were costained with antibodies against ch-TOG and acetylated α-tubulin. Centriole configuration based on

acetyl-α-tubulin labeling was used to determine cell cycle stages. Cells that had two disengaged centrioles without daughter centrioles were classified as G1, cells that had two mother centrioles each with an elongated daughter were classified as S/G2 phase. Arrowheads mark ch-TOG subdistal appendage staining, arrows point at additional ch-TOG signals including at more proximal regions. Staining with ch-TOG antibody #2 additionally labels the centriole distal ends (marked by an asterisk). One of two independent experiments shown. Scale bar, 1 µm. **b** RPE1 cells with and without microtubules were costained with antibodies against ch-TOG and acetylated α-tubulin. Scale bar, 1 µm. **c** Centriolar ch-TOG intensities were quantified, normalized to the average of the intensities in cells with microtubules, and plotted. N=2 experiments, total number of cells analyzed: 26 (MTs +) and 25 (MTs -). ** $p=0.0057$. The horizontal bars and whiskers indicate median and interquartile range, respectively, of the plotted data points. Statistical significance was determined by unpaired, two-tailed t test with Welch's correction. **d** U2OS cells were costained with antibodies against γ-tubulin and PCNT. Column 2 shows the same panels at higher exposure. Arrows point at PCM staining, arrowheads at subdistal appendage staining. Illustrations show centriole orientations. Costaining γ-tubulin/pericentrin performed once. Scale bar, 1 µm. **e** Untreated cells, cells without microtubules, and cells after microtubule regrowth for 5, 10, or 30 seconds were stained with antibodies against ch-TOG and α-tubulin to label microtubules. Insets show magnifications of regions with ch-TOG staining at centrosomes and at microtubules. Costaining performed in two independent experiments. Scale bar, 1 µm. Source data are provided as a Source Data file.

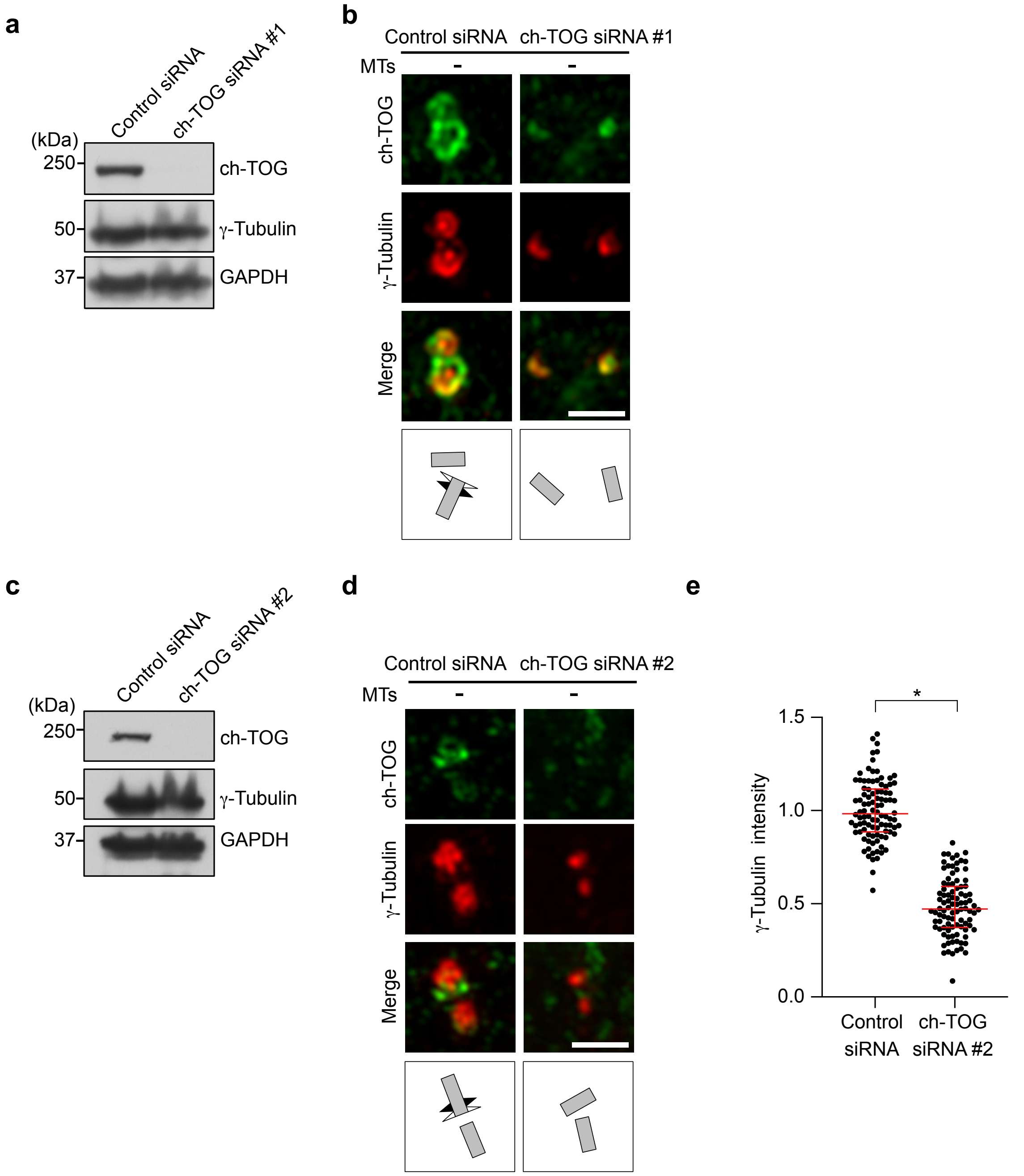

Supplementary Fig. 2. ch-TOG recruits γ-Tubulin to the centrosome. a Lysates of U2OS cells transfected with control or ch-TOG siRNA #1 were analyzed by immunoblot using antibodies against the indicated proteins. **b** Microtubules in cells transfected as in (**a**) were depolymerized and centriolar ch-TOG and γ-tubulin were revealed by staining with specific antibodies. The illustrations depict centriole configurations. **c** U2OS cell extracts after transfection of control or ch-TOG siRNA #2 were analyzed as in (**a**). **d** Analysis of centriolar ch-TOG and γ-tubulin staining of cells transfected as in (**c**) in the absence of microtubules. **e** Centriolar γ-tubulin staining was quantified in cells transfected as in (**c**), normalized to the

average of the intensities of the control, and plotted. N=2 experiments, total number of cells analyzed: 49 (Control RNAi) and 47 (ch-TOG RNAi). * p=0.0201. The horizontal bars and whiskers indicate median and interquartile range, respectively, of the plotted data points. Statistical significance was determined by unpaired, two-tailed t test with Welch's correction. Scale bar, 1 μm. Source data are provided as a Source Data file.

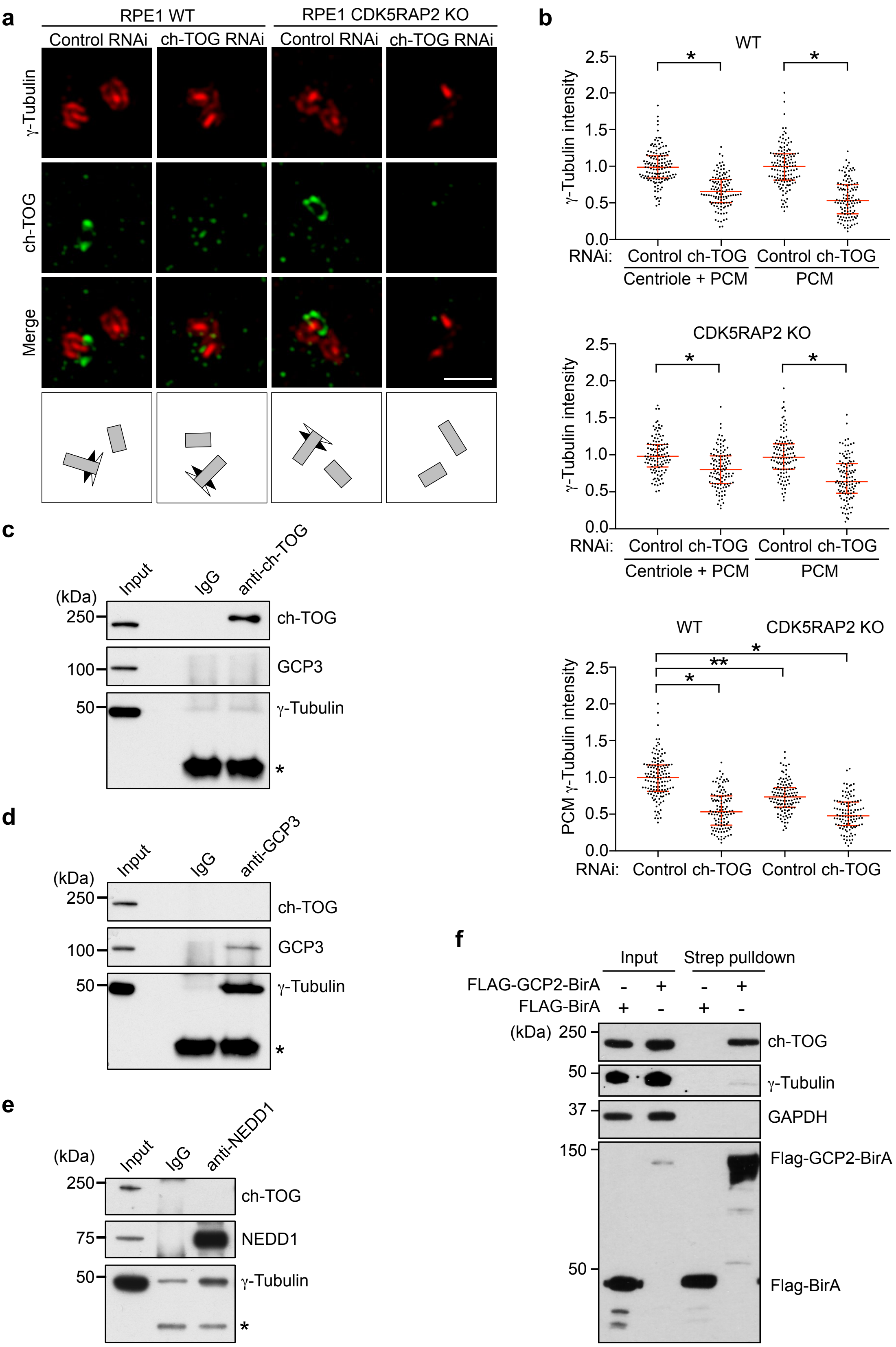

Supplementary Fig. 3. ch-TOG transiently interacts with γTuRC. a RPE1 WT or CDK5RAP2 KO cells treated with control or ch-TOG siRNA were fixed and stained with

antibodies against γ-tubulin and ch-TOG. Scale bar, 1 µm. **b** Intensities of centrosomal γ-tubulin staining in cells as in (**a**), quantified for the entire centrosome region or specifically for the PCM, were normalized to the mean of the respective control and plotted. N=3 experiments, total number of cells analyzed: 71 (WT; control RNAi), 57 (WT; ch-TOG RNAi), 69 (CDK5RAP2 KO; control RNAi), 59 (CDK5RAP2 KO; ch-TOG RNAi). *p=0.0497 (γ-tubulin at centriole + PCM in RPE1 WT cells), *p=0.0276 (γ-tubulin at PCM in RPE1 WT cells), *p=0.0422 (γ-tubulin at centriole + PCM in RPE1 CDK5RAP2 KO cells), *p=0201 (γ-tubulin at PCM in RPE1 CDK5RAP2 KO cells), *p=0.0319 (γ-tubulin at PCM in RPE1 WT vs CDK5RAP2 KO control cells), *p=0.0135 (γ-tubulin at PCM in RPE1 WT control vs CDK5RAP2 KO ch-TOG depleted cells). The horizontal bars and whiskers indicate median and interquartile range, respectively, of the plotted data points. **c, d, e** U2OS cell extracts were subjected to immunoprecipitation using antibodies against endogenous ch-TOG, GCP3, or NEDD1, as indicated. Unspecific IgG served as control. After western blotting samples were probed with antibodies against the indicated proteins. Asterisks indicate signals of antibody light chains. Each immunoprecipitation was confirmed in two independent experiments. **f** Asynchronous HEK293T cells transiently expressing Flag-BirA or Flag-GCP2-BirA and incubated with biotin for 24 hours were subjected to affinity pulldowns using streptavidin-coupled beads. After western blotting the samples were probed with antibodies to detect the indicated proteins. Detection of GAPDH served as control. The results were replicated in 2 independent experiments. Source data are provided as a Source Data file.

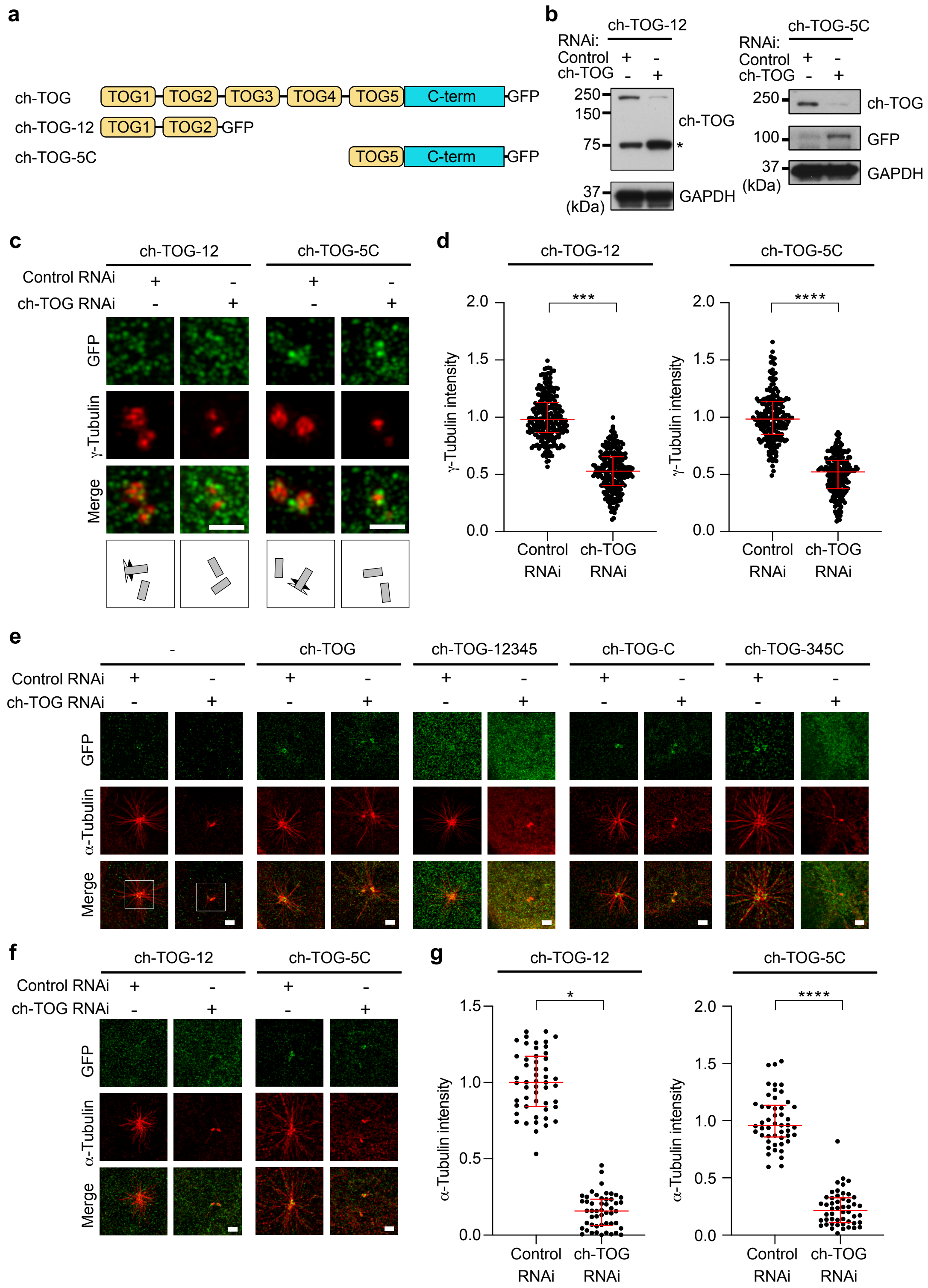

Supplementary Fig. 4. ch-TOG regulates γTuRC recruitment and microtubule nucleation. **a** Schematic representation of the domain structure of recombinant GFP-tagged full length ch-TOG and truncation mutants. TOG domains colored in yellow, C-terminal domain in cyan. **b** U2OS wild type cells and cells stably expressing recombinant GFP-tagged ch-TOG proteins as in (**a**) were transfected with control or ch-TOG siRNA #1.

Extracts were analyzed by western blotting with antibodies against the indicated proteins. The asterisk indicates the position of recombinant protein. Note that expression of recombinant proteins was always slightly elevated in conditions where endogenous ch-TOG was depleted by RNAi. **c** Cells as in (**b**) were analyzed for the centrosomal localization of recombinant ch-TOG proteins and γ-tubulin. Illustrations show centriole configurations. Scale bar, 1 µm. **d** Centriolar staining of γ-tubulin was quantified, normalized to the average of the intensities of the control, and plotted. N=4 experiments, total number of cells analyzed for control and ch-TOG RNAi, respectively: 122 and 122 (ch-TOG-12); 106 and 100 (ch-TOG-5C). **** $p<0.0001$ and **** $p<0.0001$, respectively. The horizontal bars and whiskers indicate median and interquartile range, respectively, of the plotted data points. Statistical significance was determined by unpaired, two-tailed t test with Welch's correction. **e, f** U2OS cells stably expressing the indicated recombinant ch-TOG proteins and transfected with control or ch-TOG siRNA #1 were subjected to microtubule depolymerization by incubation on ice for 30 minutes at 4°C. Following microtubule regrowth at 37°C, the cells were fixed and costained with antibodies against GFP and α-tubulin. Analyzed in two independent experiments. Scale bars, 1 µm. **g** Intensity of α-tubulin staining around centrosomes in cells as in (**f**) was quantified, normalized to the average of the intensities of the control, and plotted. N=2 experiments, total number of cells analyzed for control and ch-TOG RNAi, respectively: 51 and 50 (ch-TOG-12); 49 and 50 (ch-TOG-5C). * $p=0.0185$ (ch-TOG-12) and ** $p=0.0090$ (ch-TOG-5C), respectively. Horizontal bars and whiskers indicate median and interquartile range, respectively, of the plotted data points. Statistical significance was determined by unpaired, two-tailed t test with Welch's correction. Scale bar, 1 µm. Source data are provided as a Source Data file.

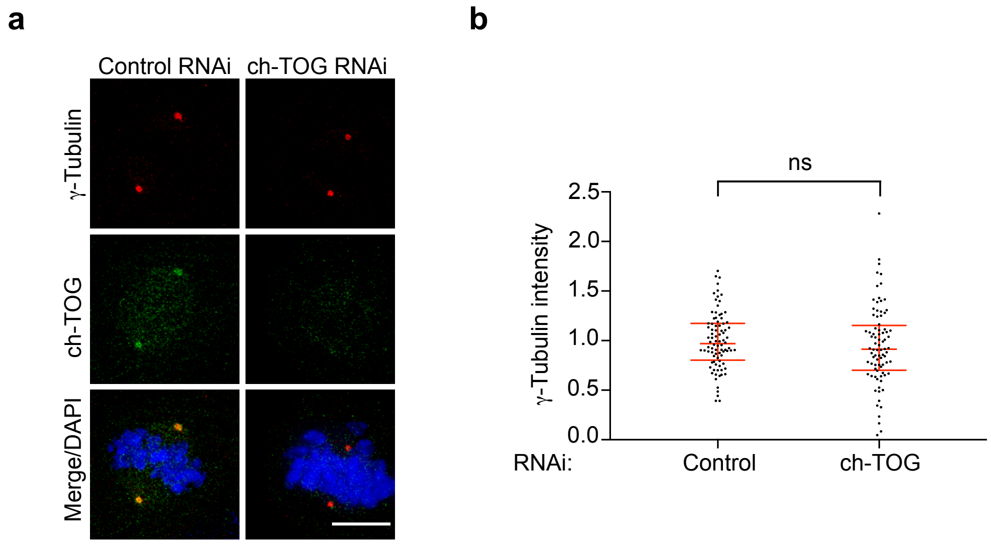

Supplementary Fig. 5. γ-Tubulin recruitment to mitotic centrosomes does not require ch-TOG. a Mitotic U2OS cells treated with control or ch-TOG siRNA were stained with γ-tubulin and ch-TOG antibodies. DAPI was used to label DNA. **b** Intensities of centrosomal γ-tubulin staining in cells as in (**a**) were normalized to the mean of the control and plotted. N=3 experiments, total number of centrosomes analyzed: 44 (Control RNAi) and 42 (ch-TOG RNAi); p=0.0893 (ns, not significant). The horizontal bars and whiskers indicate median and interquartile range, respectively, of the plotted data points. Scale bar, 2 μm. Source data are provided as a Source Data file.

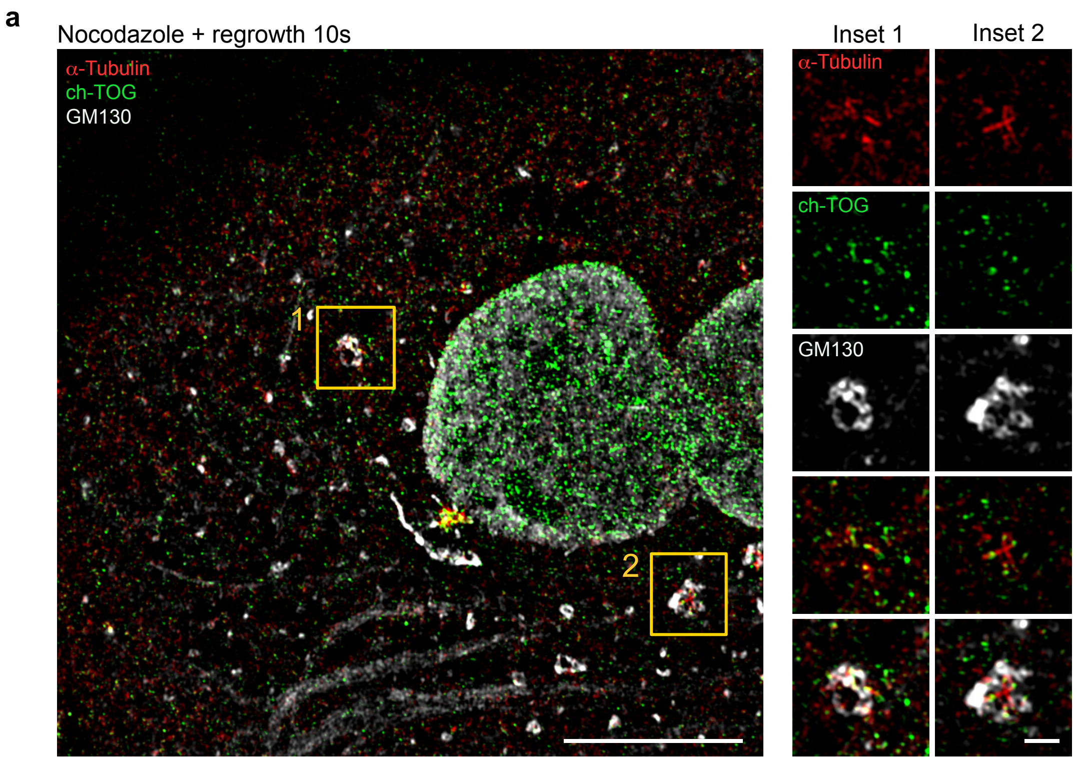

Supplementary Fig. 6. Colocalization of ch-TOG and microtubules at Golgi stacks during microtubule regrowth. RPE1 cells were treated with nocodazole to depolymerize microtubules. After washout and incubation in ice, microtubules were allowed to regrow for 10 s. Cells were fixed and stained with antibodies against GM130 (Golgi), α-tubulin (microtubules), and ch-TOG. Nucleation clusters associated with dispersed Golgi stacks are marked by yellow boxes and shown as magnifications. Similar observations were made in three independent experiments. Apart from Golgi-associated and dispersed cytoplasmic signals, the ch-TOG antibody (#1) also displayed unspecific nuclear staining. Scale bar, 10 µm; Scale bar for insets, 1 µm.
