# Supplementary material for: Microtubule nucleation and γTuRC centrosome localization in interphase cells require ch-TOG
Source: Nat Commun. 2023 Jan 26;14:289. doi: 10.1038/s41467-023-35955-w (PMC9879976; doi:10.1038/s41467-023-35955-w)
Supplement: Supplementary file 3 — Reporting Summary [file 41467_2023_35955_MOESM3_ESM.pdf]

nature portfolio

Corresponding author(s): Jens Luders

Last updated by author(s): Dec 30, 2022

Reporting Summary

Nature Portfolio wishes to improve the reproducibility of the work that we publish. This form provides structure for consistency and transparency in reporting. For further information on Nature Portfolio policies, see our Editorial Policies and the Editorial Policy Checklist.

Statistics

For all statistical analyses, confirm that the following items are present in the figure legend, table legend, main text, or Methods section.

n/a	Confirmed	
☐	☒	The exact sample size (*n*) for each experimental group/condition, given as a discrete number and unit of measurement
☐	☒	A statement on whether measurements were taken from distinct samples or whether the same sample was measured repeatedly
☐	☒	The statistical test(s) used AND whether they are one- or two-sided<br>*Only common tests should be described solely by name; describe more complex techniques in the Methods section.*
☒	☐	A description of all covariates tested
☒	☐	A description of any assumptions or corrections, such as tests of normality and adjustment for multiple comparisons
☐	☒	A full description of the statistical parameters including central tendency (e.g. means) or other basic estimates (e.g. regression coefficient) AND variation (e.g. standard deviation) or associated estimates of uncertainty (e.g. confidence intervals)
☐	☒	For null hypothesis testing, the test statistic (e.g. *F*, *t*, *r*) with confidence intervals, effect sizes, degrees of freedom and *P* value noted<br>*Give P values as exact values whenever suitable.*
☒	☐	For Bayesian analysis, information on the choice of priors and Markov chain Monte Carlo settings
☒	☐	For hierarchical and complex designs, identification of the appropriate level for tests and full reporting of outcomes
☒	☐	Estimates of effect sizes (e.g. Cohen's *d*, Pearson's *r*), indicating how they were calculated

Our web collection on statistics for biologists contains articles on many of the points above.

Software and code

Policy information about availability of computer code

Data collection	Zen black 2.3 (Zeiss)
Data analysis	Zen black 2.3 (Zeiss); Fiji 2.9.0 (Open Source); Prism 9.4.0 (Graphpad)

For manuscripts utilizing custom algorithms or software that are central to the research but not yet described in published literature, software must be made available to editors and reviewers. We strongly encourage code deposition in a community repository (e.g. GitHub). See the Nature Portfolio guidelines for submitting code & software for further information.

Data

Policy information about availability of data

All manuscripts must include a data availability statement. This statement should provide the following information, where applicable:

- Accession codes, unique identifiers, or web links for publicly available datasets
- A description of any restrictions on data availability
- For clinical datasets or third party data, please ensure that the statement adheres to our policy

All data generated or analysed during this study, including source data for the presented results, are included in the article and its supplementary information files.

Human research participants

Policy information about studies involving human research participants and Sex and Gender in Research.

Reporting on sex and gender	n/a
Population characteristics	n/a
Recruitment	n/a
Ethics oversight	n/a

Note that full information on the approval of the study protocol must also be provided in the manuscript.

Field-specific reporting

Please select the one below that is the best fit for your research. If you are not sure, read the appropriate sections before making your selection.

☒ Life sciences ☐ Behavioural & social sciences ☐ Ecological, evolutionary & environmental sciences

For a reference copy of the document with all sections, see nature.com/documents/nr-reporting-summary-flat.pdf

Life sciences study design

All studies must disclose on these points even when the disclosure is negative.

Sample size	Sample sizes were determined based on experience from previous, similar analyses and based on methods used in related published studies (e.g. Gavilan et al, 2018, EMBO reports 19:e45942; Wu et al., 2016, Developmental Cell 39, 44–60)
Data exclusions	No data were excluded from the analysis.
Replication	Data were replicated in at least 2 independent experiments with similar results. The exact number of replicates is indicated for each experiment in the respective figure legend.
Randomization	In this study no animal or human subjects were analyzed. Cells were randomly allocated to culture wells and cells treated with specific siRNAs or expressing specific cDNA were randomly allocated to each group.
Blinding	Investigators were not blinded to group allocations. In all experiments groups were discernible by obvious differences in immunofluorescence staining or western blot band patterns and in some cases this was required for analysis, which made blinding not feasible.

Reporting for specific materials, systems and methods

We require information from authors about some types of materials, experimental systems and methods used in many studies. Here, indicate whether each material, system or method listed is relevant to your study. If you are not sure if a list item applies to your research, read the appropriate section before selecting a response.

Materials & experimental systems

n/a	Involved in the study	
☐	☒	Antibodies
☐	☒	Eukaryotic cell lines
☒	☐	Palaeontology and archaeology
☒	☐	Animals and other organisms
☒	☐	Clinical data
☒	☐	Dual use research of concern

Methods

n/a	Involved in the study	
☒	☐	ChIP-seq
☒	☐	Flow cytometry
☒	☐	MRI-based neuroimaging

Antibodies

Antibodies used	Primary antibodies IF: ch-TOG (#1, 1:100, Abcam, ab86073), ch-TOG (#2, 1:250, Santa Cruz Biotechnology, sc-374394), CEP128 (1:250, Bethyl, A303-348A), GFP (1:500, Thermo Fisher Scientific, A-6455), ODF2 (1:500, Abcam, ab43840), Ac-alpha-Tubulin (1:500, SIGMA, T6793), gamma-Tubulin (1:500, EXBIO, 11-4645-C100), Ninein (1:500, EMD Millipore Corp, MABT29), PCNT (1:500; Luders et al., 2006, Nat Cell Biol 8, 137–147; Tim Stearns, Stanford University, USA), NEDD1 (1:500 ; Luders et al., 2006, Nat Cell Biol 8, 137–

147; Tim Stearns, Stanford University, USA), alpha-Tubulin (1:500, SIMGA T6199), alpha-Tubulin (1:250, Abcam, ab18251).
Secondary antibodies IF: Alexa-Fluor-488-, Alexa-Fluor-568- and Alexa-Fluor-647-conjugated, cross-adsorbed secondary goat anti-rabbit or goat anti-mouse antibodies (1:500; Thermo Fisher).
Primary antibodies Western: ch-TOG (#1, 1:1000, Abcam ab86073), ch-TOG (#2, 1:1000, Santa Cruz Biotechnology sc-374394), gamma-Tubulin (1:3000, SIGMA T6557), GFP (1:2000, Torrey Pines Biolabs TP401), FLAG (1:10000, SIGMA F1804), GCP3 (1:2000; Cota et al., 2017, J Cell Sci 130, 406–419; Jens Lüders, IRB Barcelona, Spain), NEDD1 (1:2000; Luders et al., 2006, Nat Cell Biol 8, 137–147; Tim Stearns, Stanford University; USA), GAPDH (1:10000, Santa Cruz Biotechnology sc-47724).
Secondary antibodies Western: HRP-coupled goat anti-rabbit and goat anti-mouse antibodies (1:5000; Jackson ImmunoResearch Laboratories, AB_10015289, AB_2313567).

Validation

For two different commercial antibodies (ch-TOG #1, Abcam, ab86073), ch-TOG #2, Santa Cruz Biotechnology, sc-374394) as well as all custom-made antibodies (PCNT; NEDD1; GCP3) we validated the specificity of the signals in both immunofluorescence microscopy and western blot by RNAi-mediated depletion (ch-TOG #1, ch-TOG #2: this manuscript; PCNT, NEDD1: Luders et al., 2006, Nat Cell Biol 8, 137–147; Haren et al., 2009, Plos One, 4, e5976; GCP3: Cota et al., 2017, J Cell Sci 130, 406–419. For commercial antibodies CEP128 (Bethyl, A303-348A), ODF2 (Abcam, ab43840), Ninein (EMD Millipore Corp, MABT29) the specificity of centriole labeling was confirmed by RNAi and/or colocalization with known markers (this manuscript). For the remaining commercial antibodies we did not perform specific validation, but only used reagents that are commonly used and established in the field. All antibodies produced the staining patterns in cells and/or band sizes in western blots that were as expected based on the manufacturer's information and based on numerous previous studies.

Eukaryotic cell lines

Policy information about cell lines and Sex and Gender in Research

Cell line source(s)

HEK293T, U2OS, hTERT RPE1 (ATCC); hTERT RPE1 CDK5RAP2 KO (Wu et al., 2016, Developmental Cell 39, 44–60; Anna Akhmanova, Utrecht University, The Netherlands)

Authentication

Cell lines were not authenticated.

Mycoplasma contamination

All cell lines tested negative for mycoplasma contamination.

Commonly misidentified lines
(See ICLAC register)

No commonly misidentified cell line was used.
