# Supplementary figures and images for: Microtubule nucleation and γTuRC centrosome localization in interphase cells require ch-TOG
Source: Nat Commun. 2023 Jan 26;14:289. doi: 10.1038/s41467-023-35955-w (PMC9879976; doi:10.1038/s41467-023-35955-w)

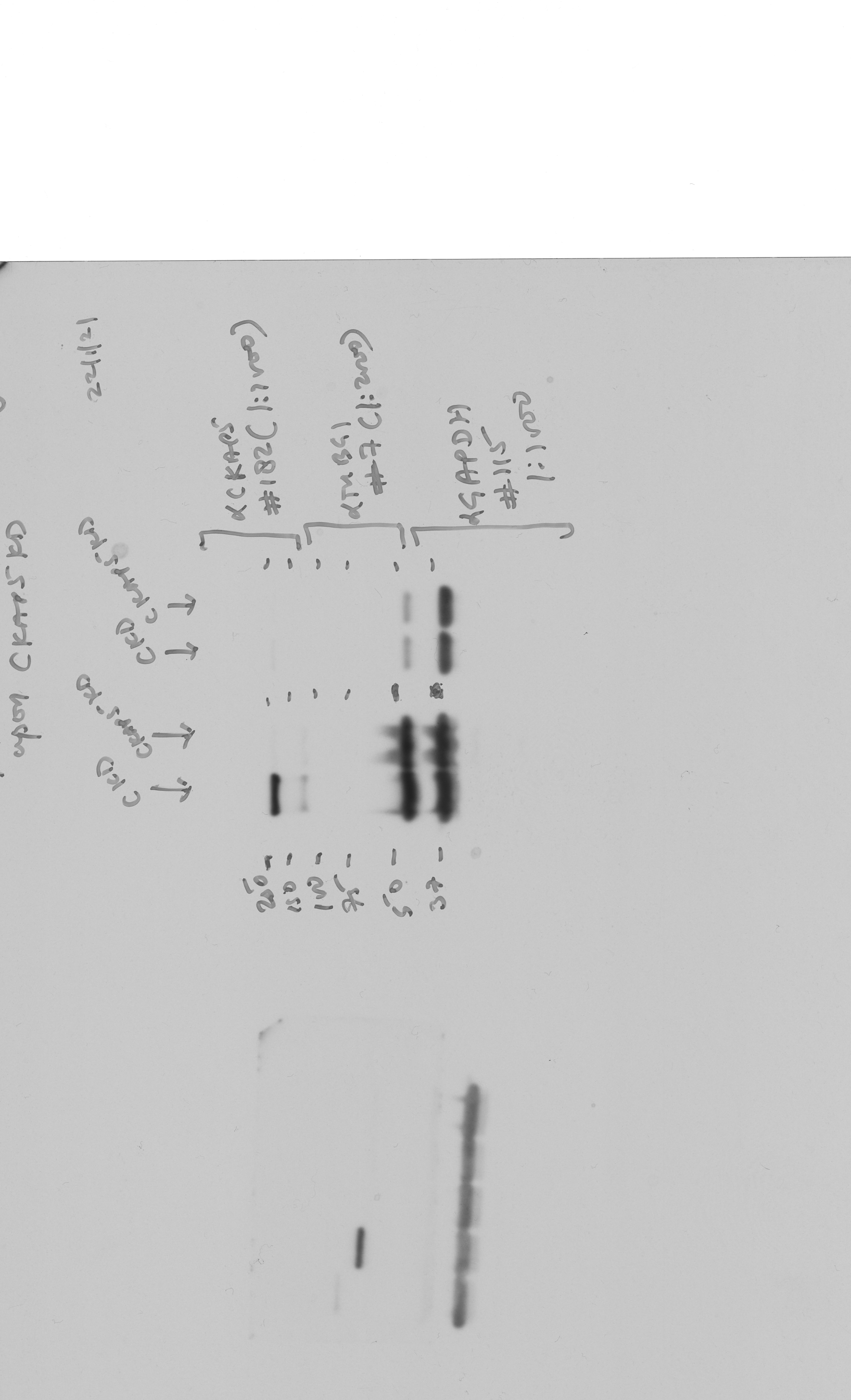

Supplement: Supplementary file 4 — Source Data [file 41467_2023_35955_MOESM4_ESM.zip › Source data_2ndrev_JL/Uncropped Western scans/Supplementary Figure 2A/Supplementary Fig 2A_ckap5 kd sirna 1 22-1-21013.tif]

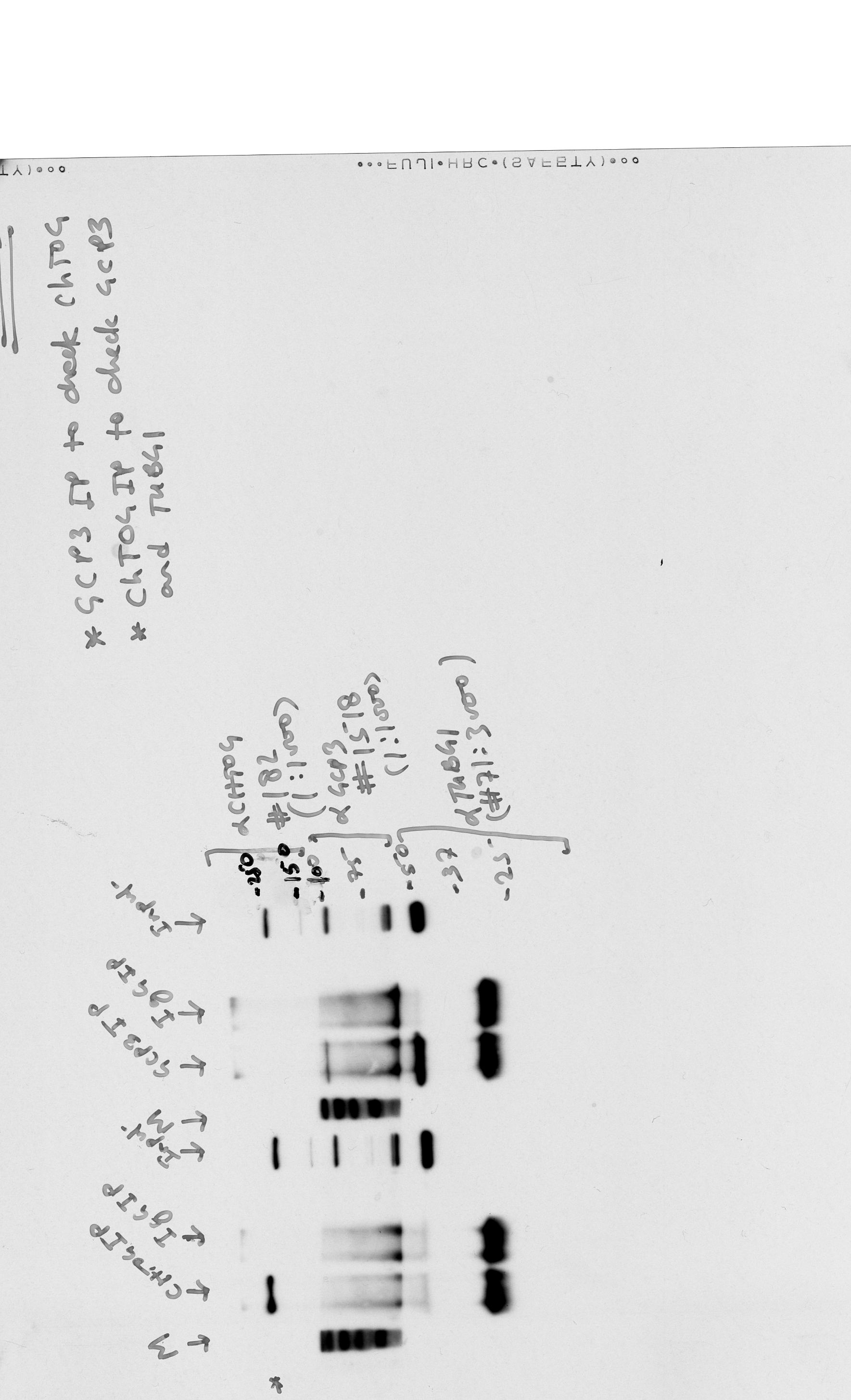

Supplement: Supplementary file 4 — Source Data [file 41467_2023_35955_MOESM4_ESM.zip › Source data_2ndrev_JL/Uncropped Western scans/Supplementary Figure 3C and 3D/Supplementary Fig 3C and 3D_ckap5 and gcp3 IP17-6-20010.tif]

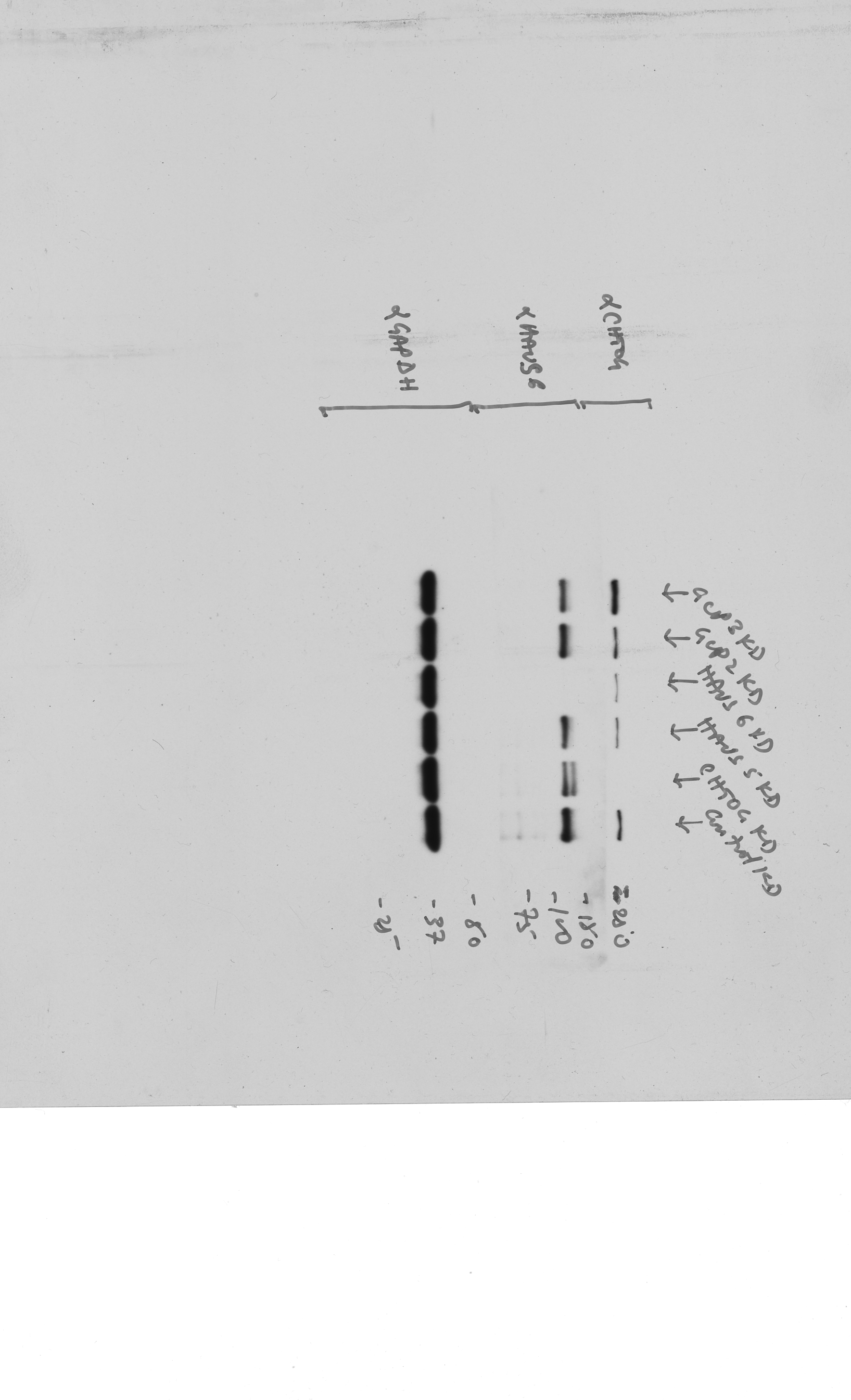

Supplement: Supplementary file 4 — Source Data [file 41467_2023_35955_MOESM4_ESM.zip › Source data_2ndrev_JL/Uncropped Western scans/Figure 1B/Fig 1B_c and ckap5 KD 2018.tif]

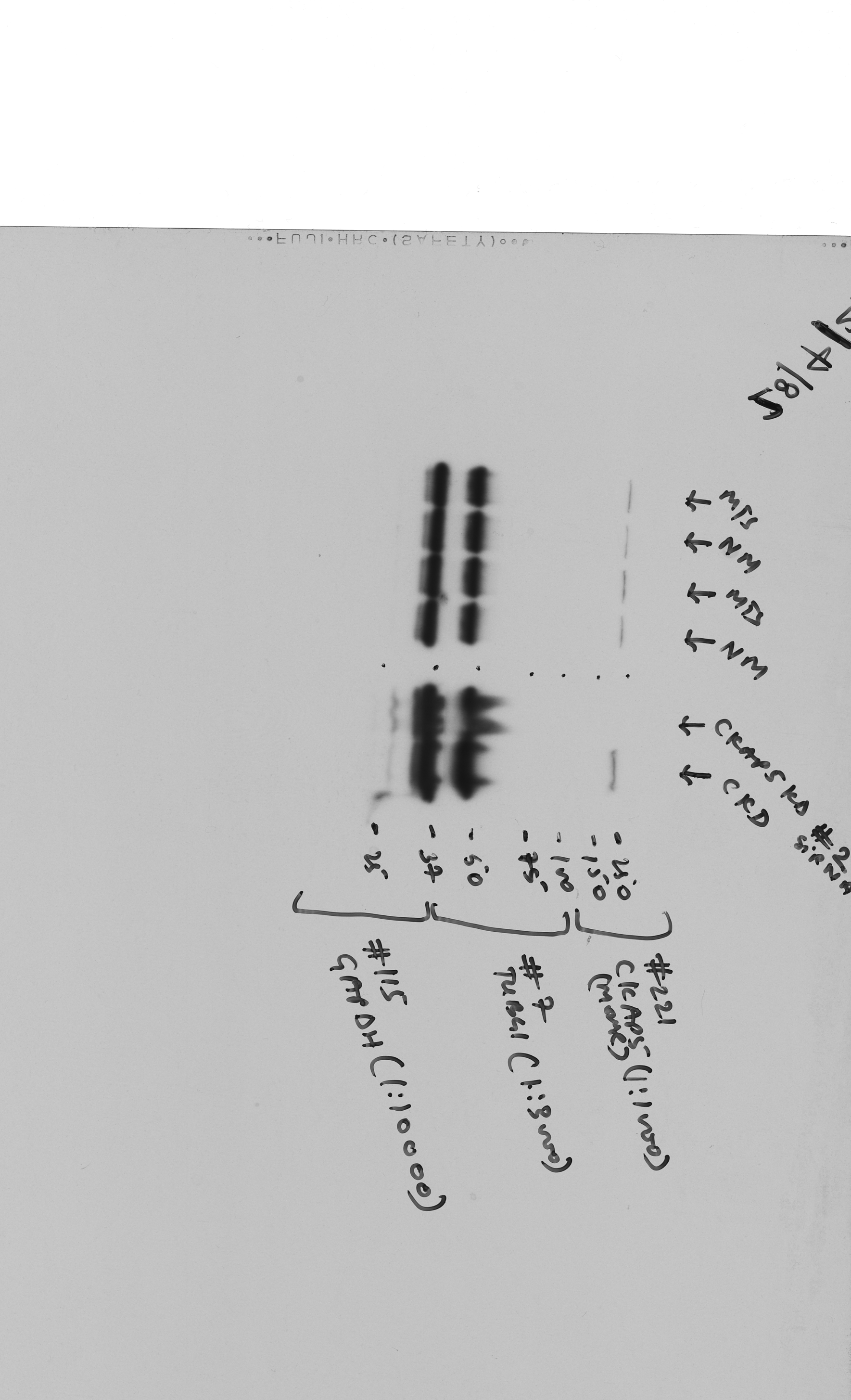

Supplement: Supplementary file 4 — Source Data [file 41467_2023_35955_MOESM4_ESM.zip › Source data_2ndrev_JL/Uncropped Western scans/Figure 2D/Fig 2D_ckap5 kd sirna 2 28-4-21 exposure 1.tif]

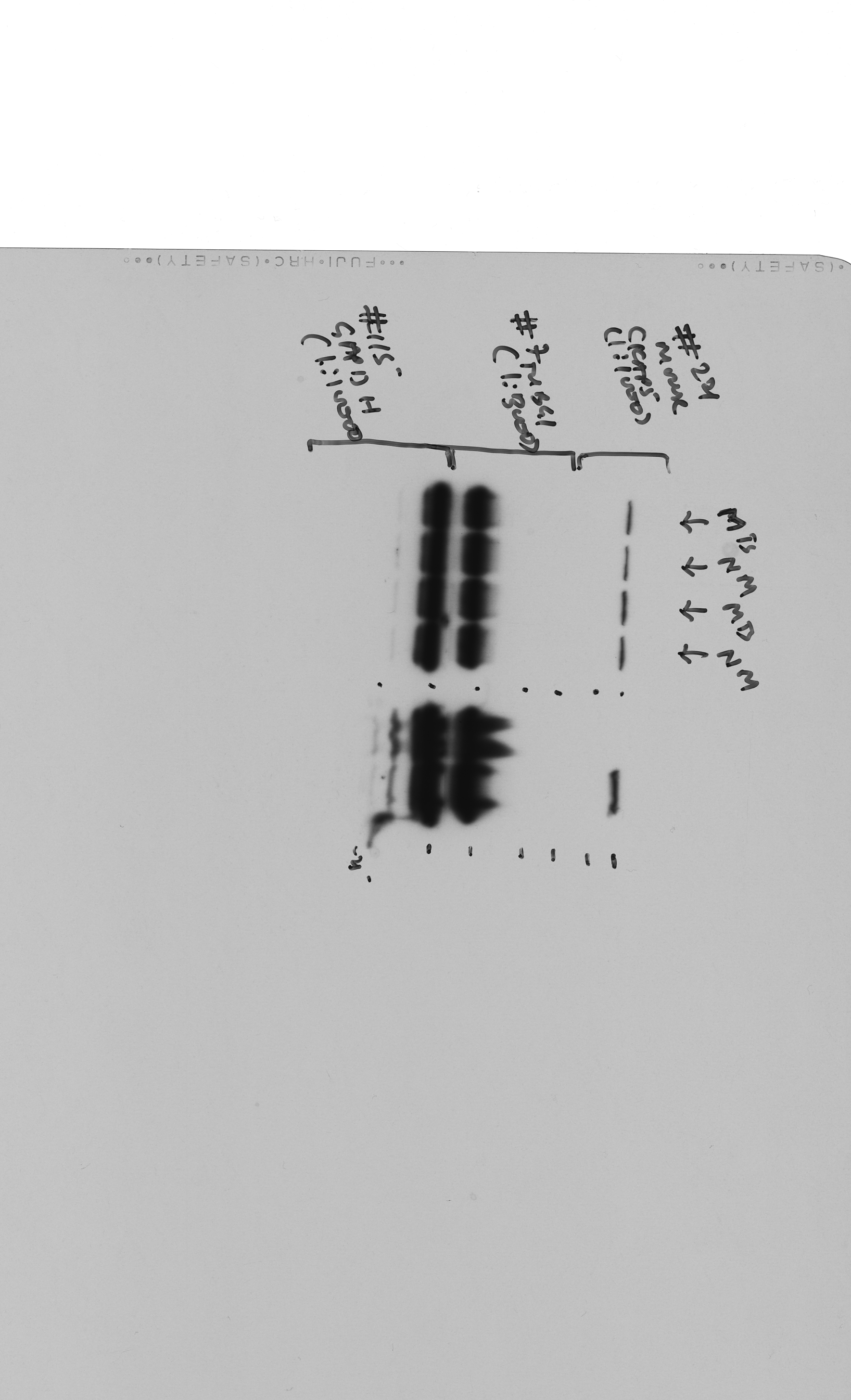

Supplement: Supplementary file 4 — Source Data [file 41467_2023_35955_MOESM4_ESM.zip › Source data_2ndrev_JL/Uncropped Western scans/Figure 2D/Fig 2D_ckap5 kd sirna 2 28-4-21 exposure 2.tif]

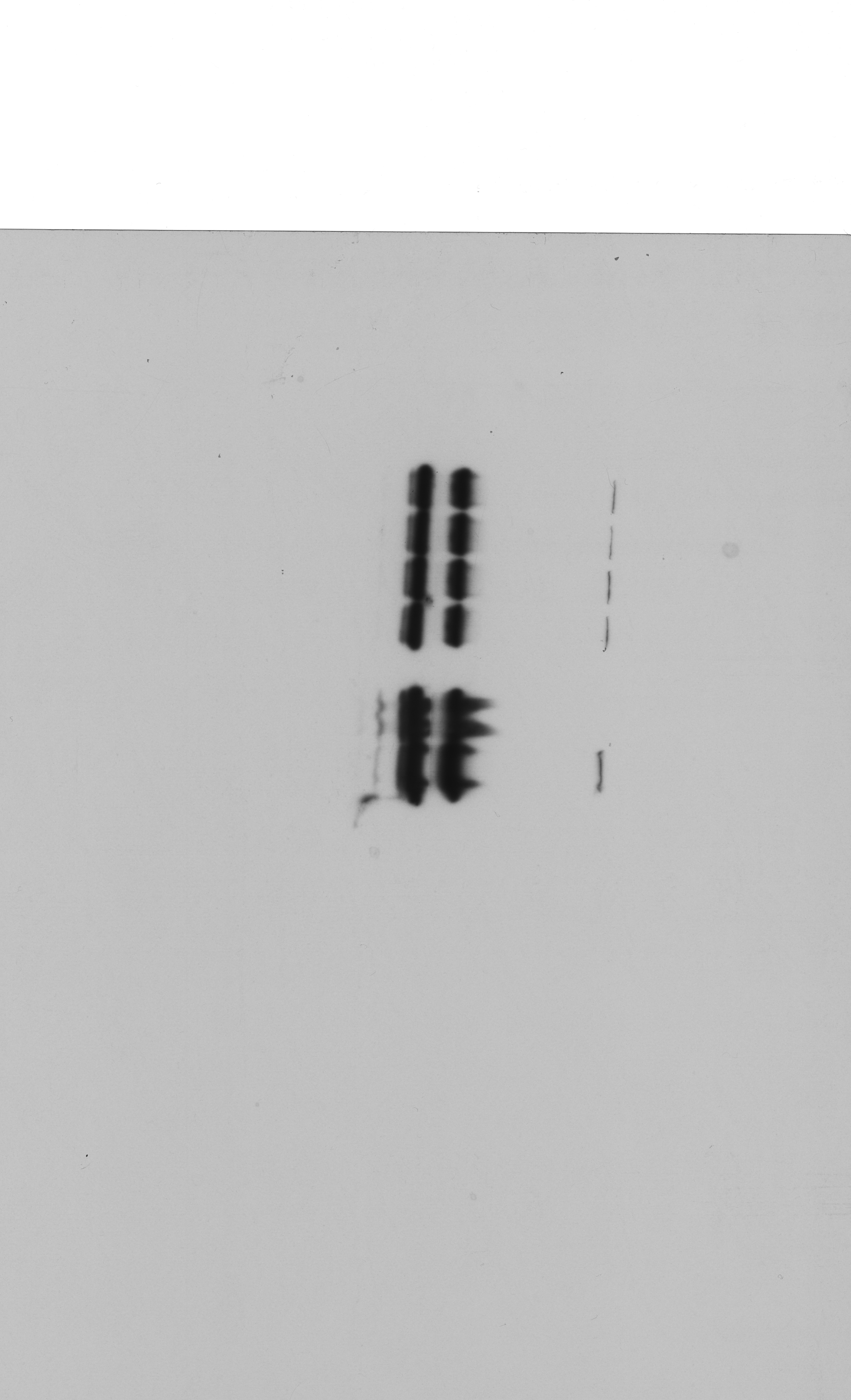

Supplement: Supplementary file 4 — Source Data [file 41467_2023_35955_MOESM4_ESM.zip › Source data_2ndrev_JL/Uncropped Western scans/Supplementary Figure 2C/Supplementary Fig 2C_ckap5 kd sirna 2 28-4-21 exposure 3.tif]

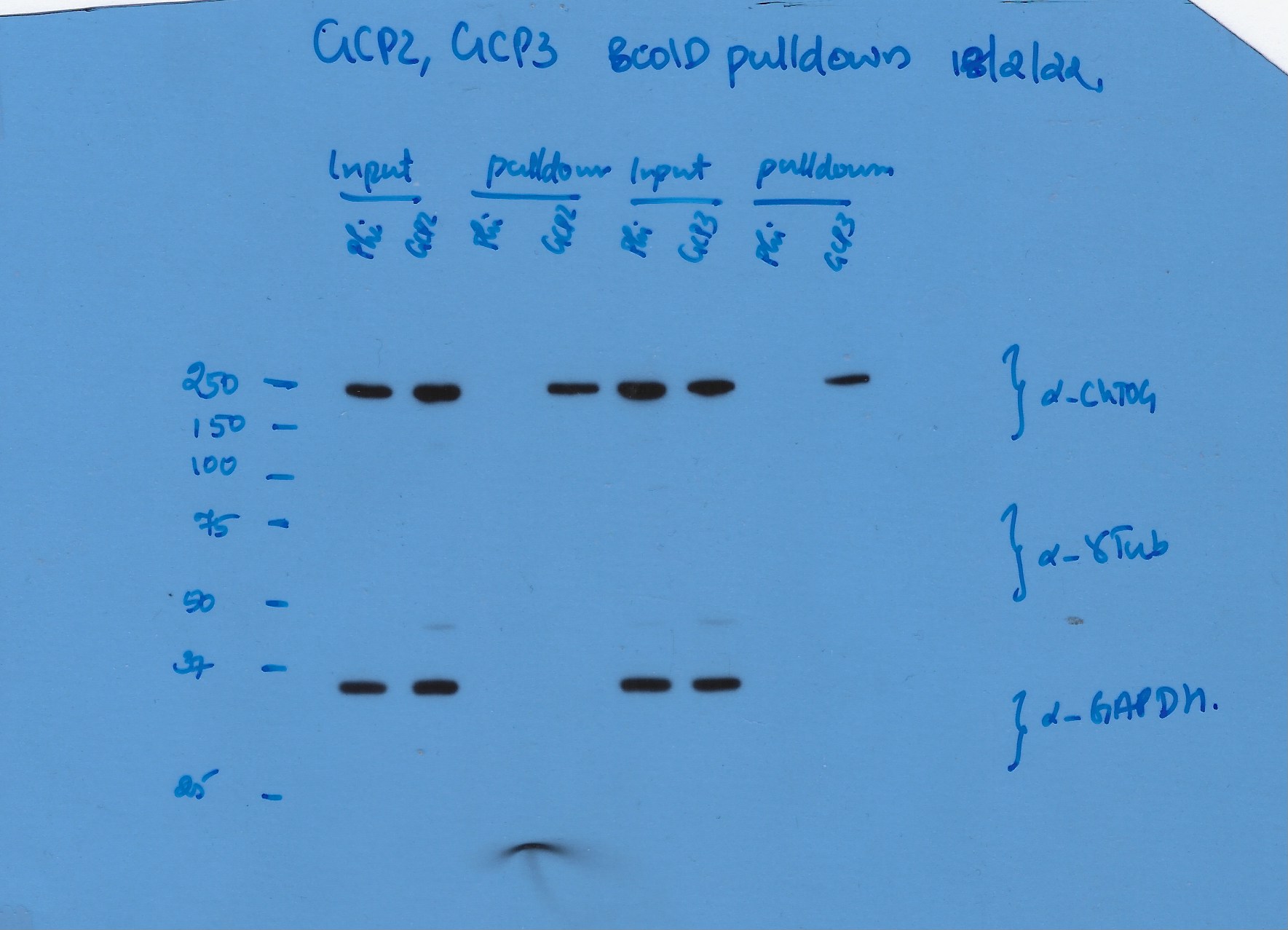

Supplement: Supplementary file 4 — Source Data [file 41467_2023_35955_MOESM4_ESM.zip › Source data_2ndrev_JL/Uncropped Western scans/Figure 3G /Fig3G_Trial 1/Fig3G_BirAGCP3 BioID pulldown trial 1_CKAP5, GAPDH.jpeg]

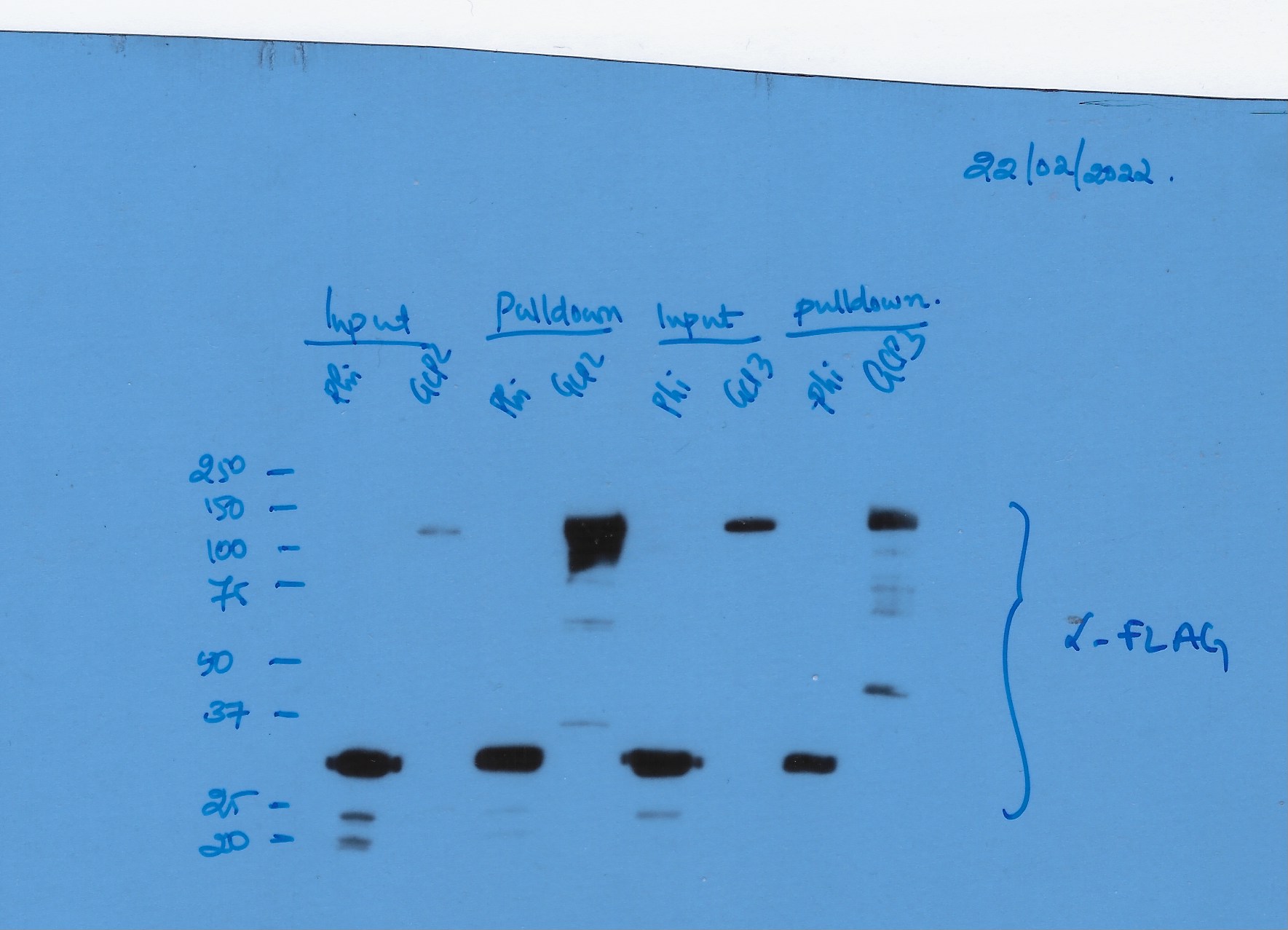

Supplement: Supplementary file 4 — Source Data [file 41467_2023_35955_MOESM4_ESM.zip › Source data_2ndrev_JL/Uncropped Western scans/Figure 3G /Fig3G_Trial 1/Fig3G_BirAGCP3 BioID pulldown trial 1_FLAG.jpeg]

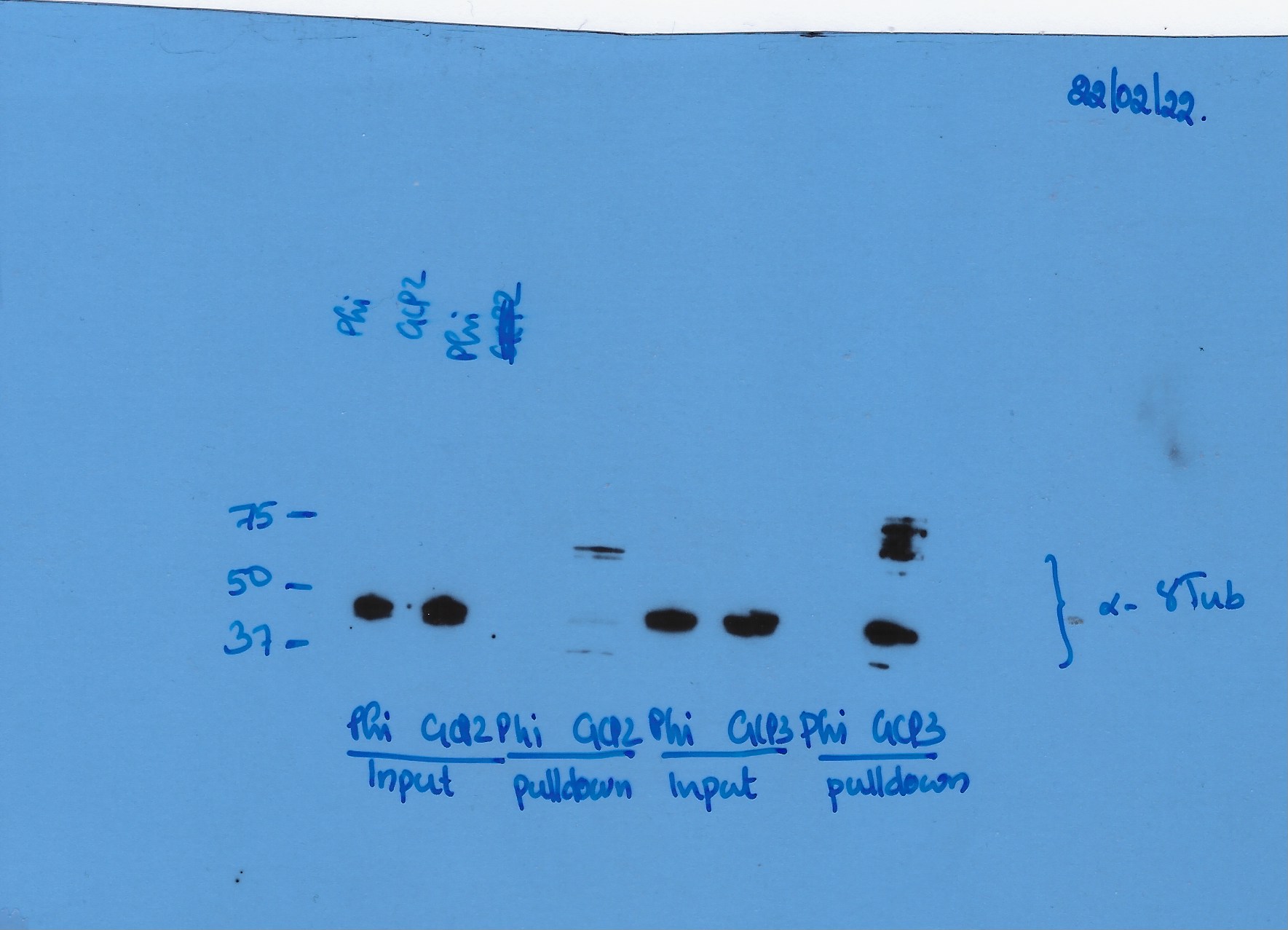

Supplement: Supplementary file 4 — Source Data [file 41467_2023_35955_MOESM4_ESM.zip › Source data_2ndrev_JL/Uncropped Western scans/Figure 3G /Fig3G_Trial 1/Fig3G_BirAGCP3 BioID pulldown trial 1_gTub.jpeg]

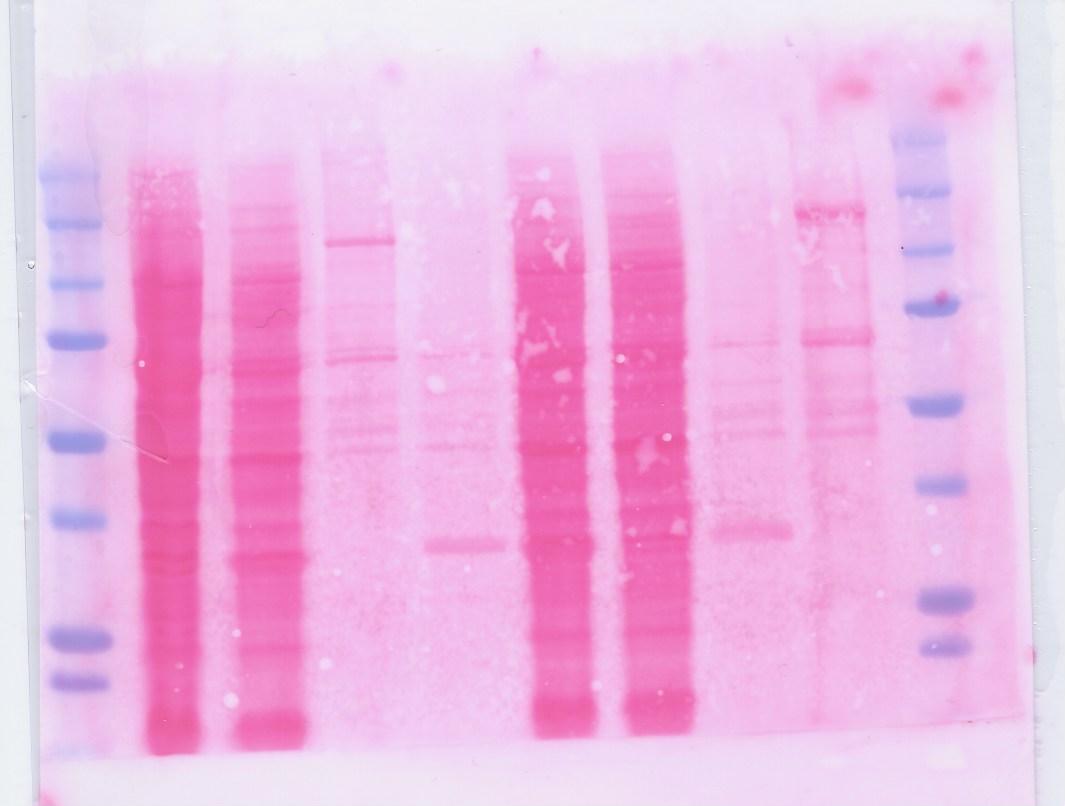

Supplement: Supplementary file 4 — Source Data [file 41467_2023_35955_MOESM4_ESM.zip › Source data_2ndrev_JL/Uncropped Western scans/Figure 3G /Fig3G_Trial 1/Fig3G_BirAGCP3 BioID pulldown trial 1_Ponceau.jpeg]

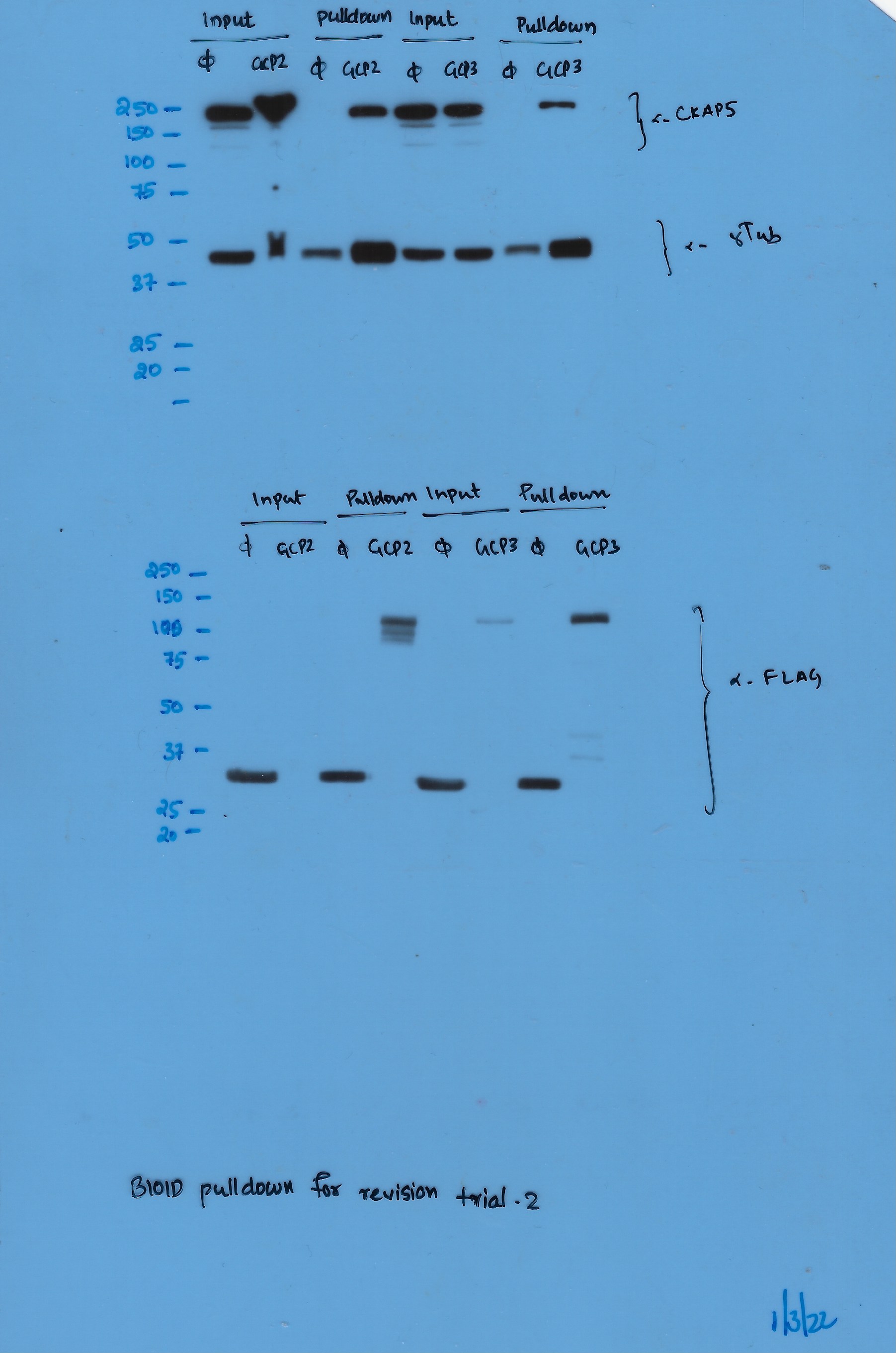

Supplement: Supplementary file 4 — Source Data [file 41467_2023_35955_MOESM4_ESM.zip › Source data_2ndrev_JL/Uncropped Western scans/Figure 3G /Fig3G_Trial 2/Fig3G_BirAGCP3 BioID pulldown trial 2_CKAP5 FLAG gTub.jpeg]

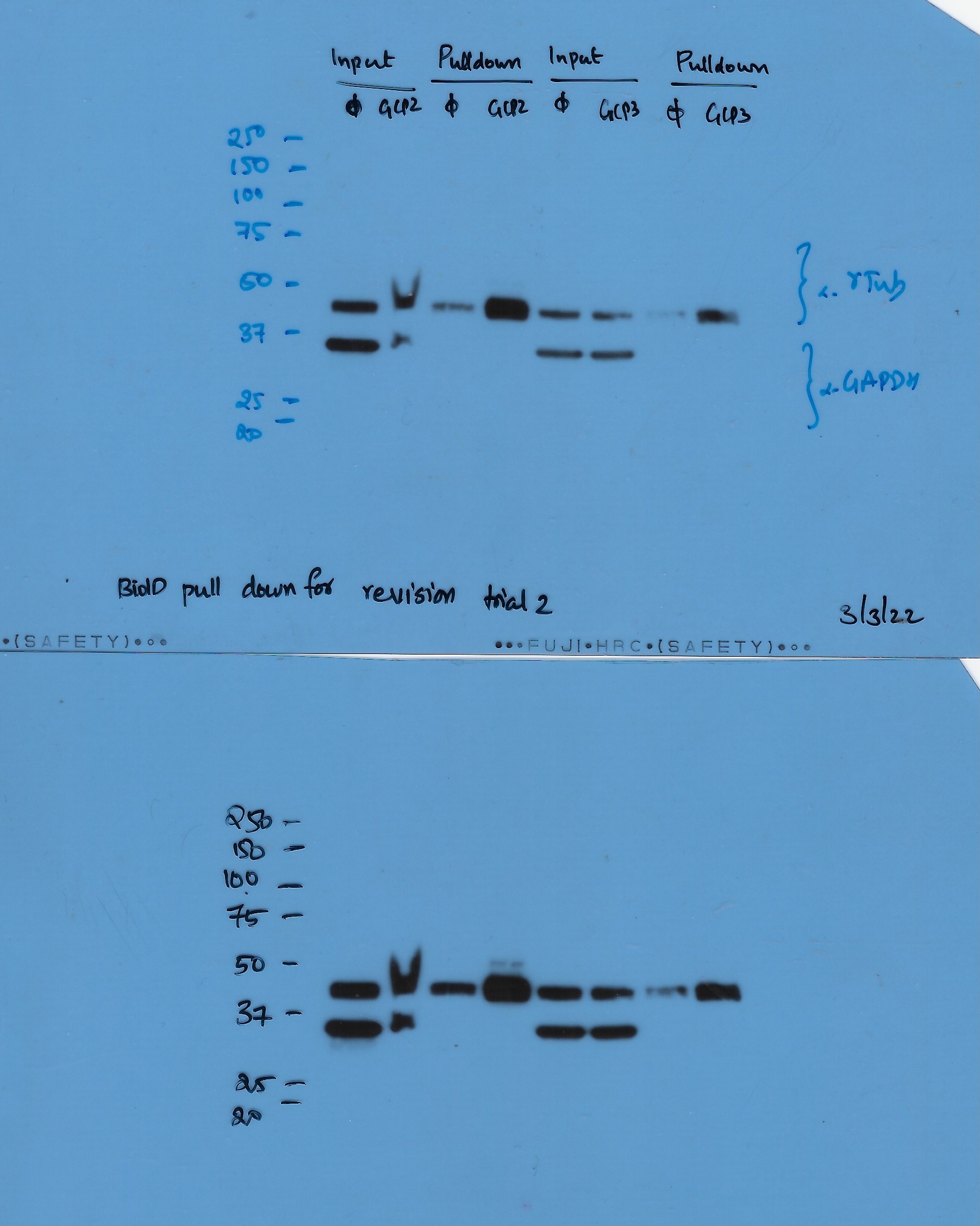

Supplement: Supplementary file 4 — Source Data [file 41467_2023_35955_MOESM4_ESM.zip › Source data_2ndrev_JL/Uncropped Western scans/Figure 3G /Fig3G_Trial 2/Fig3G_BirAGCP3 BioID pulldown trial 2_gTub GAPDH.jpeg]

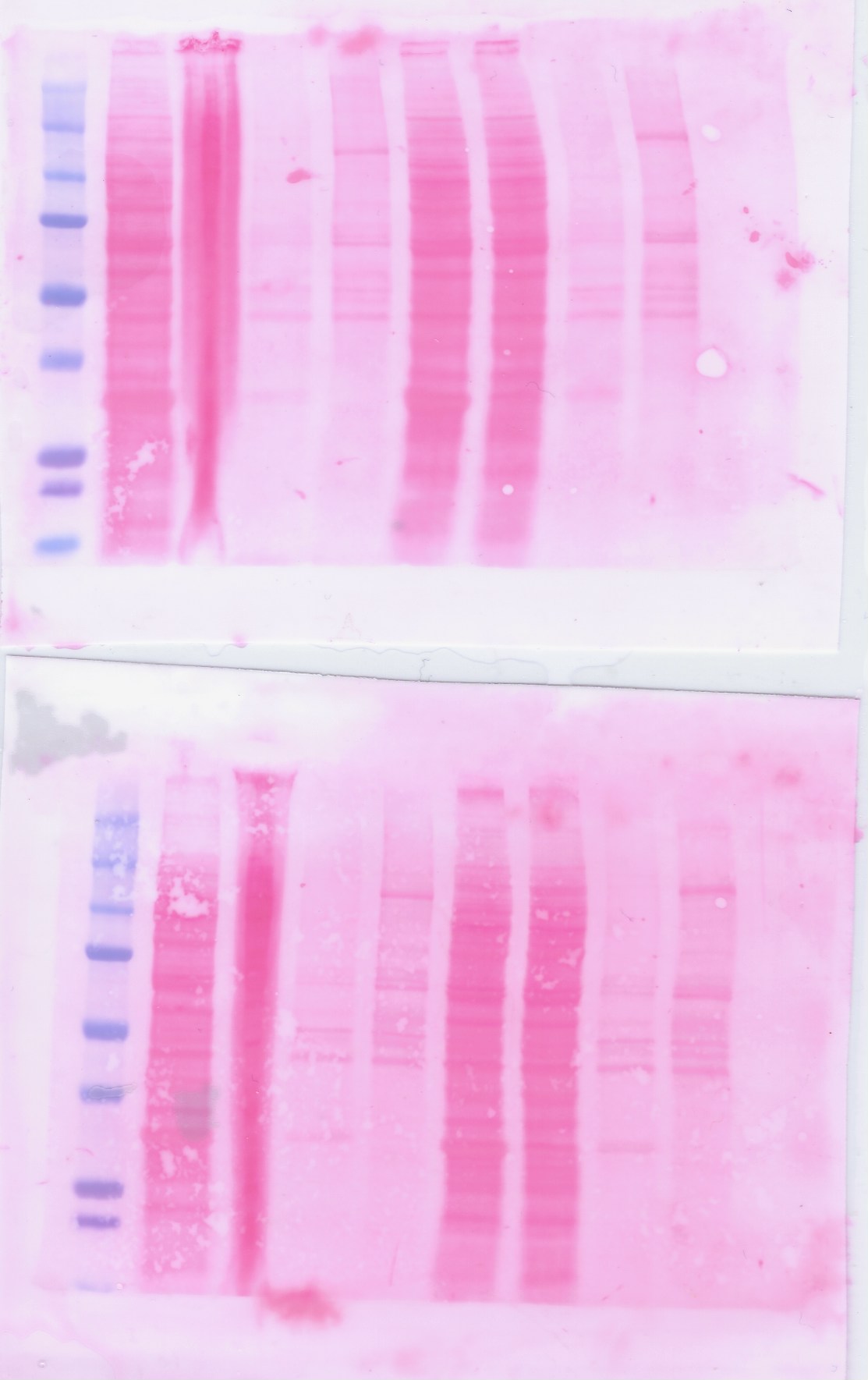

Supplement: Supplementary file 4 — Source Data [file 41467_2023_35955_MOESM4_ESM.zip › Source data_2ndrev_JL/Uncropped Western scans/Figure 3G /Fig3G_Trial 2/Fig3G_BirAGCP3 BioID pulldown trial 2_ponceau.jpeg]

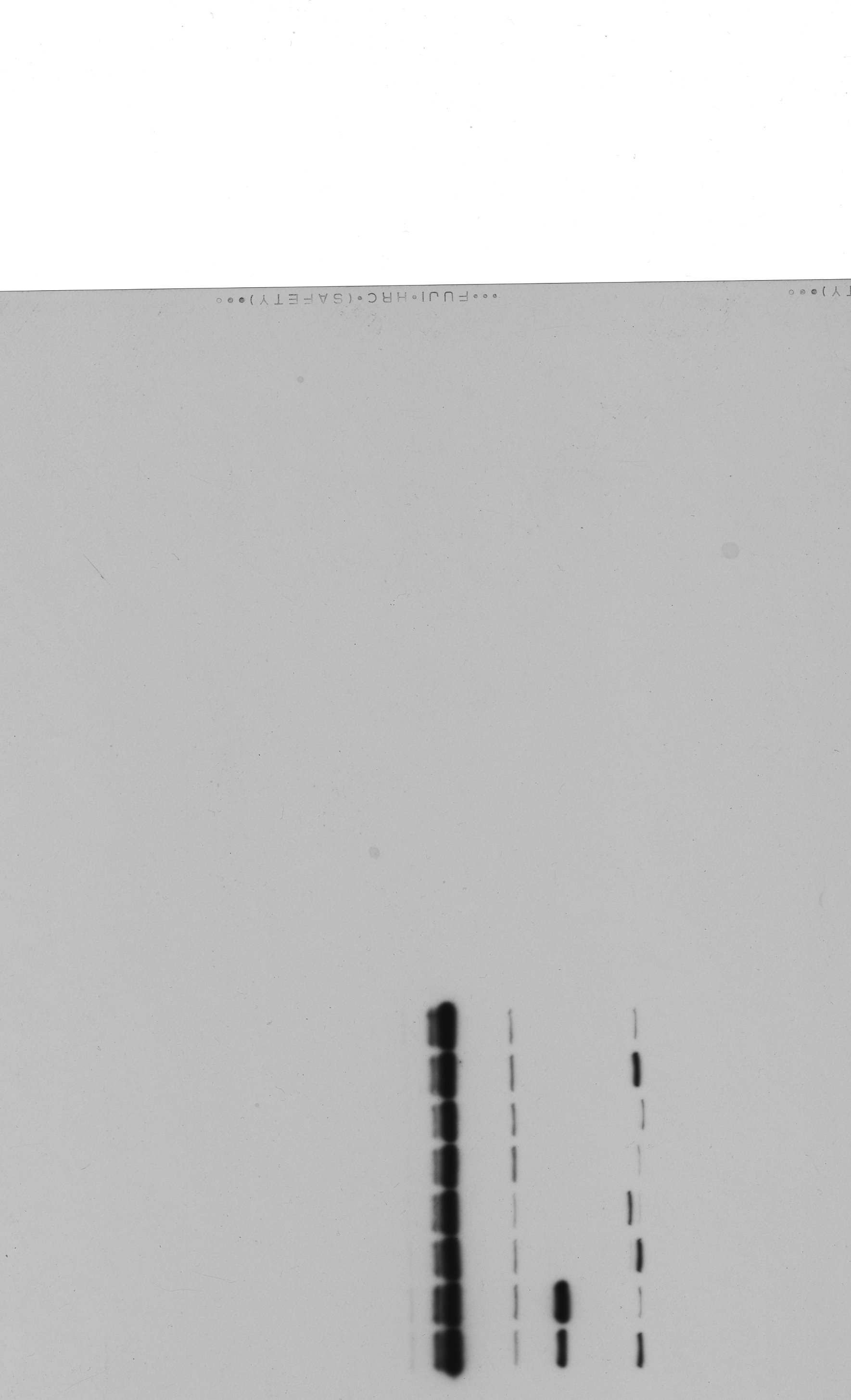

Supplement: Supplementary file 4 — Source Data [file 41467_2023_35955_MOESM4_ESM.zip › Source data_2ndrev_JL/Uncropped Western scans/Figure 5B/Fig 5B_CKAP5 KD in - CKAP5 CKAP5-12 CKAP5-12345 constructs/Fig 5B_ckap5 kd in (-) CKAP5-FL CKAP5-12 CKAP5-12345 constructs 31-3-21 exposure 3.tif]

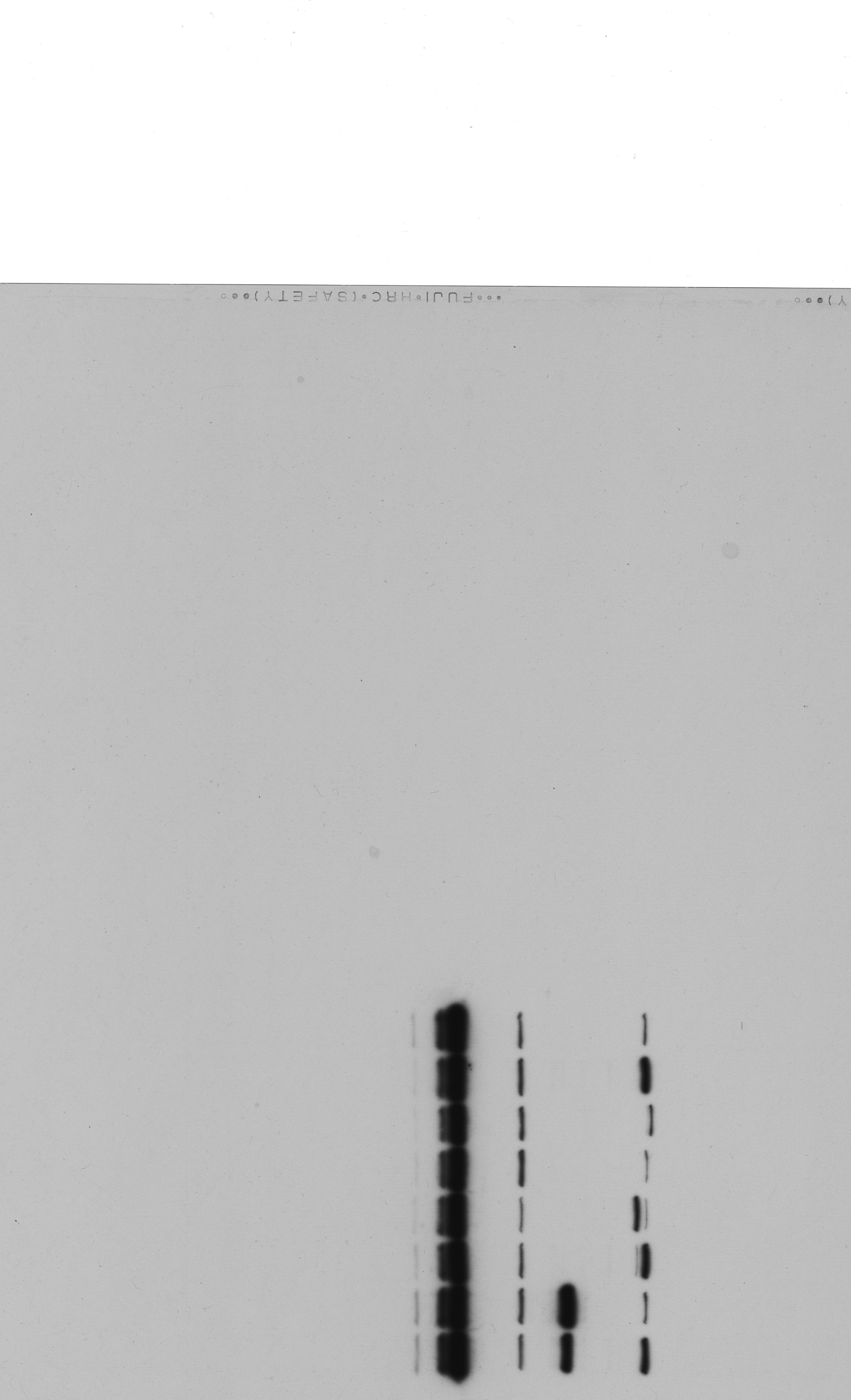

Supplement: Supplementary file 4 — Source Data [file 41467_2023_35955_MOESM4_ESM.zip › Source data_2ndrev_JL/Uncropped Western scans/Figure 5B/Fig 5B_CKAP5 KD in - CKAP5 CKAP5-12 CKAP5-12345 constructs/Fig 5B_ckap5 kd in (-) CKAP5-FL CKAP5-12 CKAP5-12345 constructs 31-3-21 exposure 2.tif]

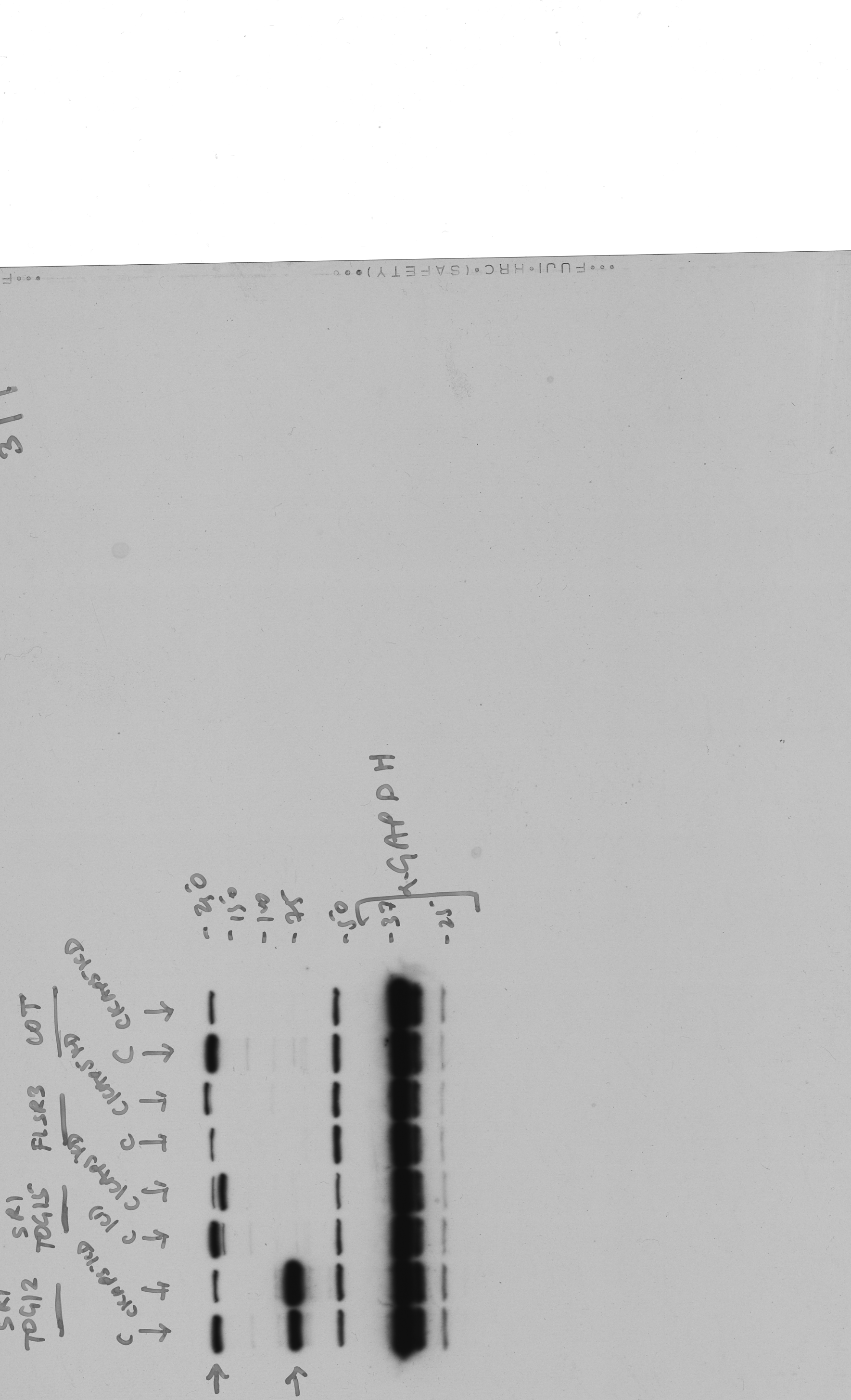

Supplement: Supplementary file 4 — Source Data [file 41467_2023_35955_MOESM4_ESM.zip › Source data_2ndrev_JL/Uncropped Western scans/Figure 5B/Fig 5B_CKAP5 KD in - CKAP5 CKAP5-12 CKAP5-12345 constructs/Fig 5B_ckap5 kd in (-) CKAP5-FL CKAP5-12 CKAP5-12345 constructs 31-3-21 exposure 1.tif]

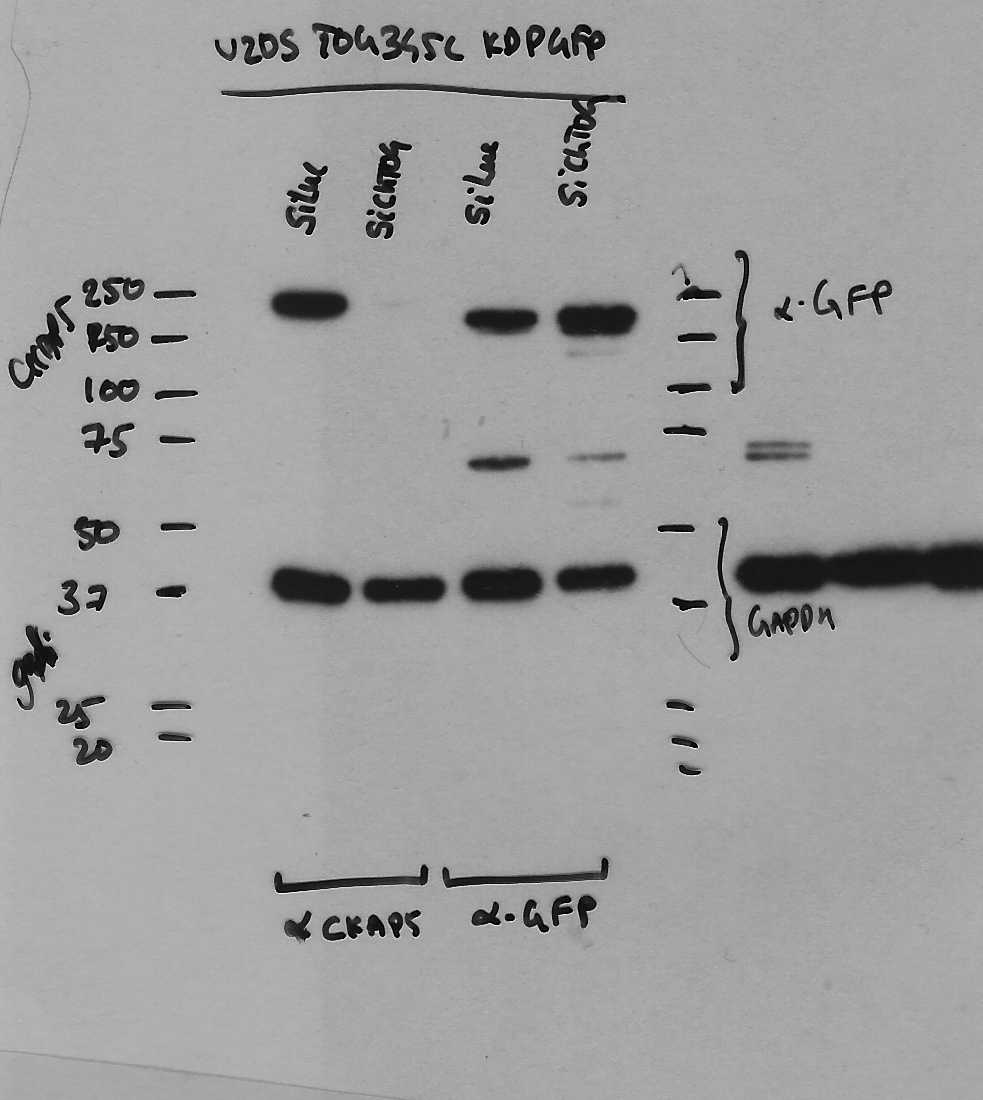

Supplement: Supplementary file 4 — Source Data [file 41467_2023_35955_MOESM4_ESM.zip › Source data_2ndrev_JL/Uncropped Western scans/Figure 5B/Fig 5B_CKAP5 KD in CKAP5-345C construct/Fig 5B_CKAP5 KD in CKAP5-345C construct exposure 1.tiff]

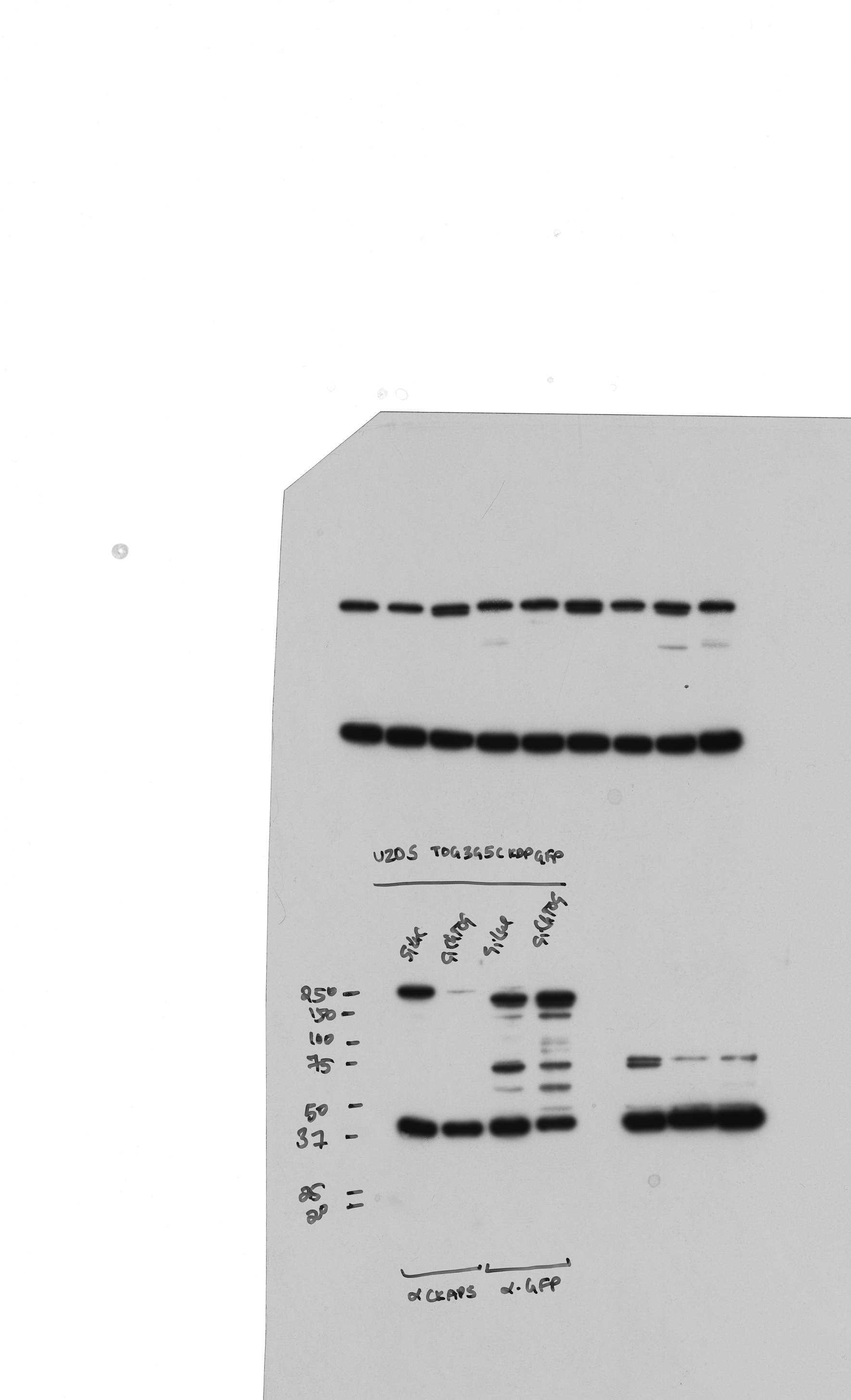

Supplement: Supplementary file 4 — Source Data [file 41467_2023_35955_MOESM4_ESM.zip › Source data_2ndrev_JL/Uncropped Western scans/Figure 5B/Fig 5B_CKAP5 KD in CKAP5-345C construct/Fig 5B_CKAP5 KD in CKAP5-345C construct exposure 2.tif]

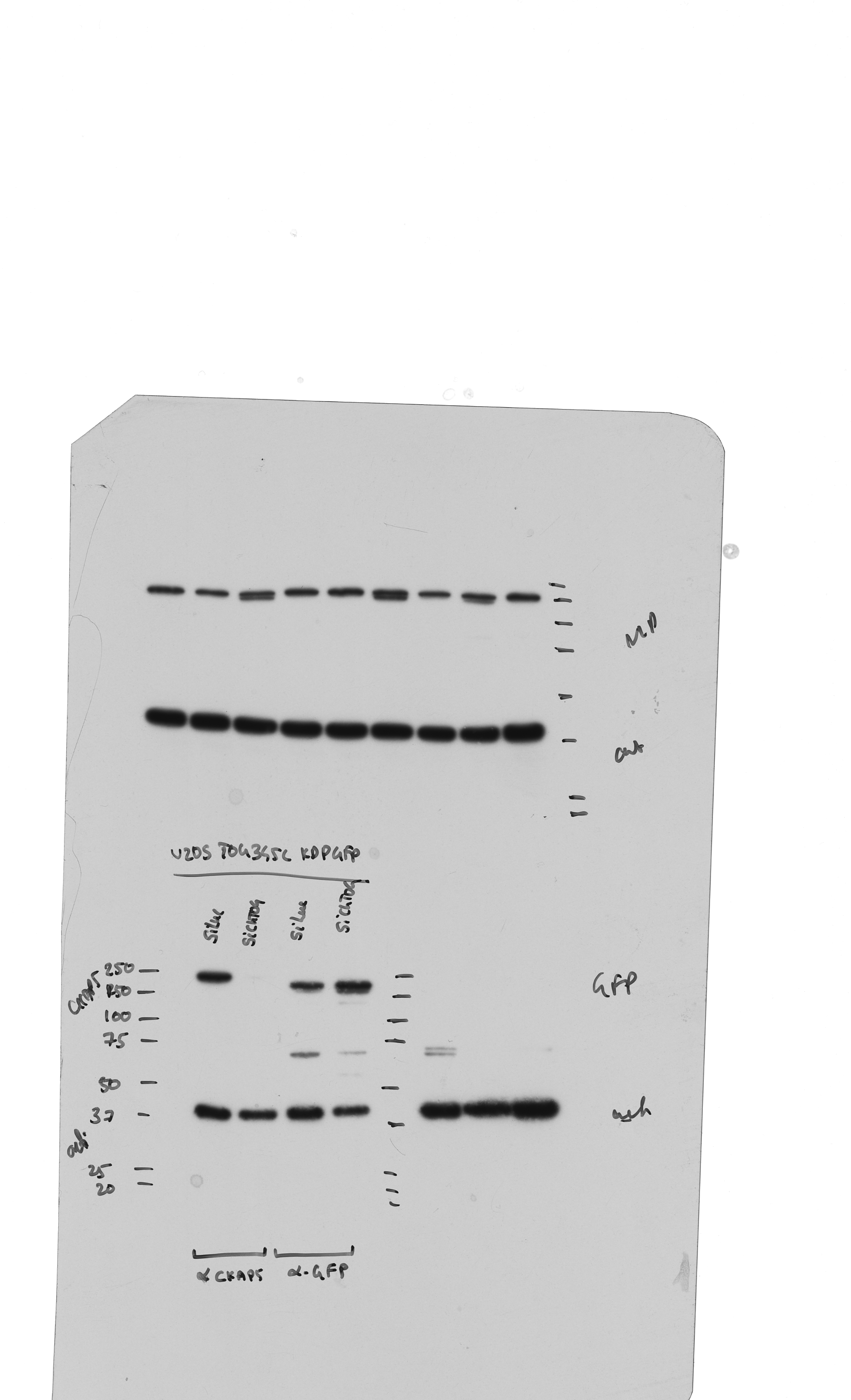

Supplement: Supplementary file 4 — Source Data [file 41467_2023_35955_MOESM4_ESM.zip › Source data_2ndrev_JL/Uncropped Western scans/Figure 5B/Fig 5B_CKAP5 KD in CKAP5-345C construct/Fig 5B_CKAP5 KD in CKAP5-345C construct exposure 3.tif]

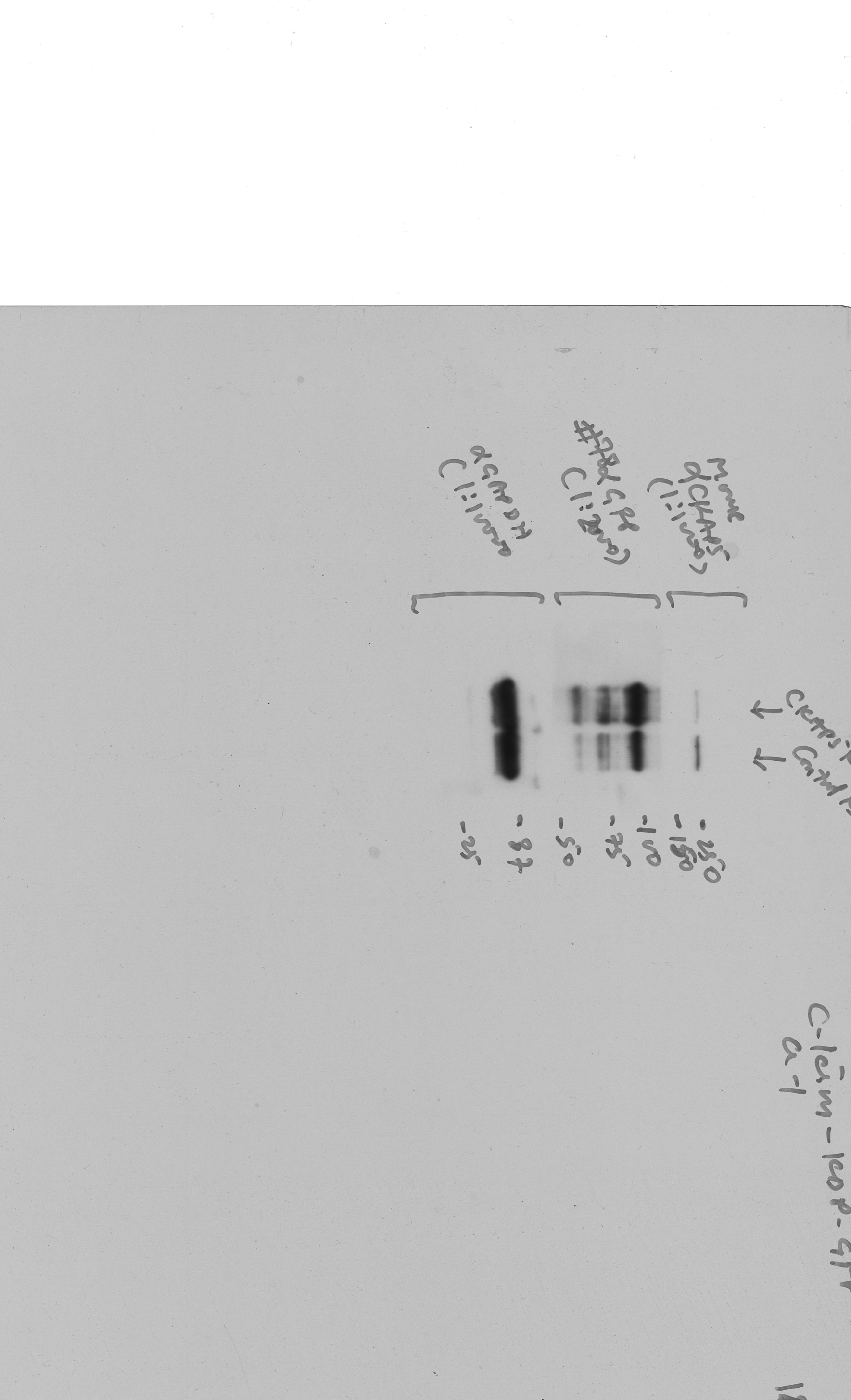

Supplement: Supplementary file 4 — Source Data [file 41467_2023_35955_MOESM4_ESM.zip › Source data_2ndrev_JL/Uncropped Western scans/Figure 5B/Fig 5B_CKAP5 KD in CKAP5-C construct/Fig 5B_ckap5 kd in CKAP5-C construct 14-4-21.tif]

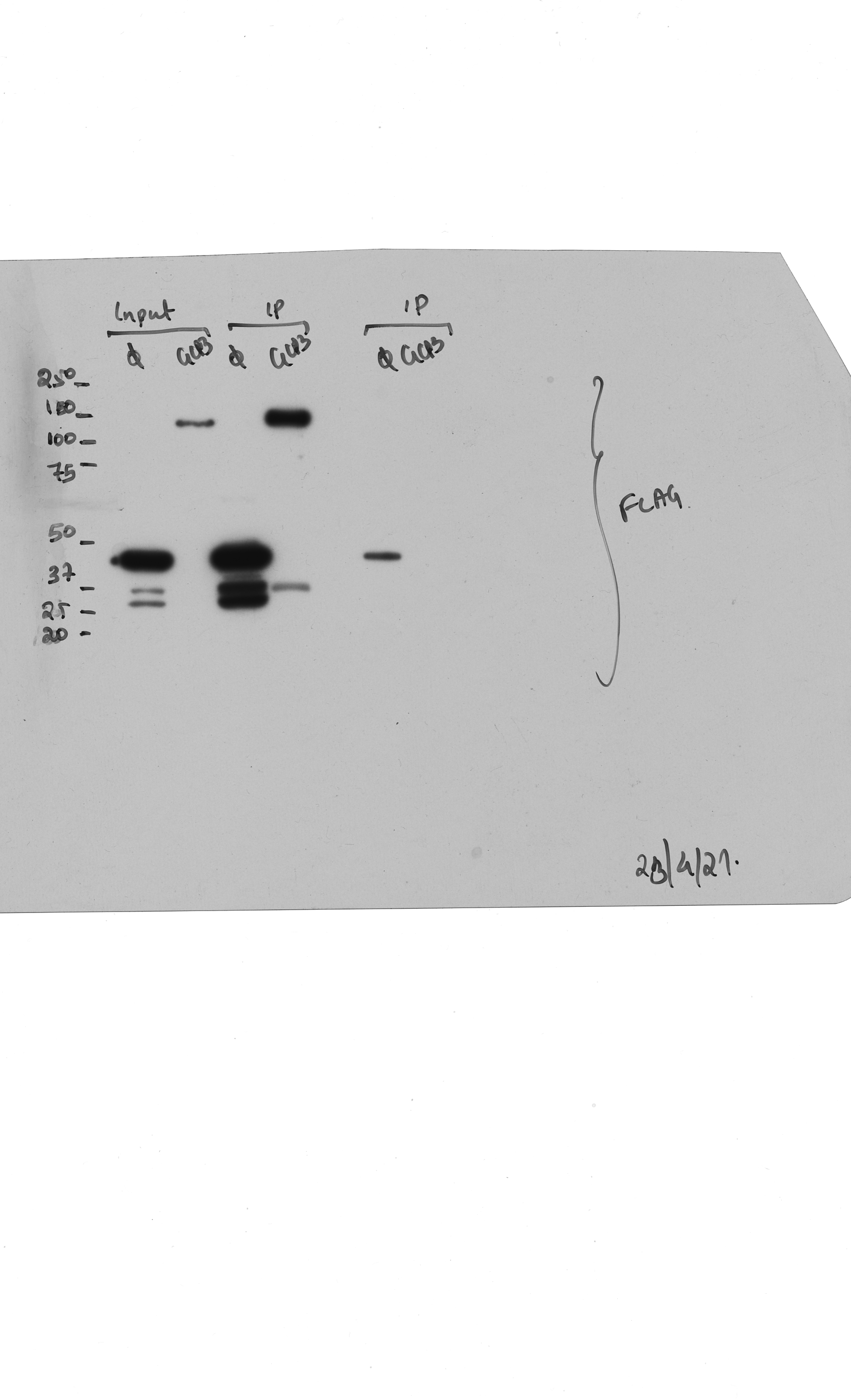

Supplement: Supplementary file 4 — Source Data [file 41467_2023_35955_MOESM4_ESM.zip › Source data_2ndrev_JL/Uncropped Western scans/Figure 3F/Fig3F_FLAG IP with GCP3-BirA construct/Fig3F_FLAG immunoblot GCP3 bira flag ip 2.tif]

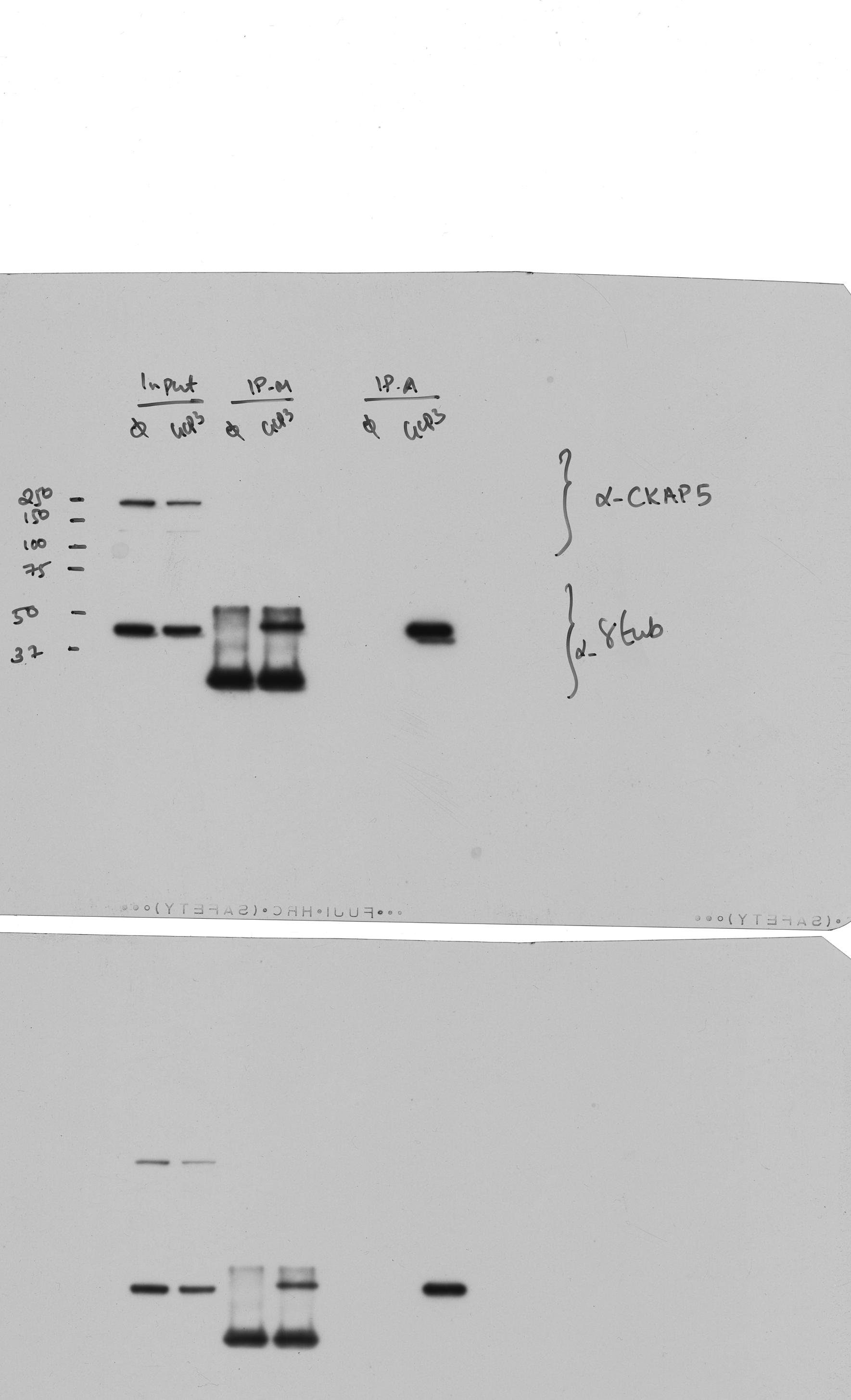

Supplement: Supplementary file 4 — Source Data [file 41467_2023_35955_MOESM4_ESM.zip › Source data_2ndrev_JL/Uncropped Western scans/Figure 3F/Fig3F_FLAG IP with GCP3-BirA construct/Fig3F_Anti CKAP5 and anti yTUB immunoblot GCP3 bira flag IP.tif]

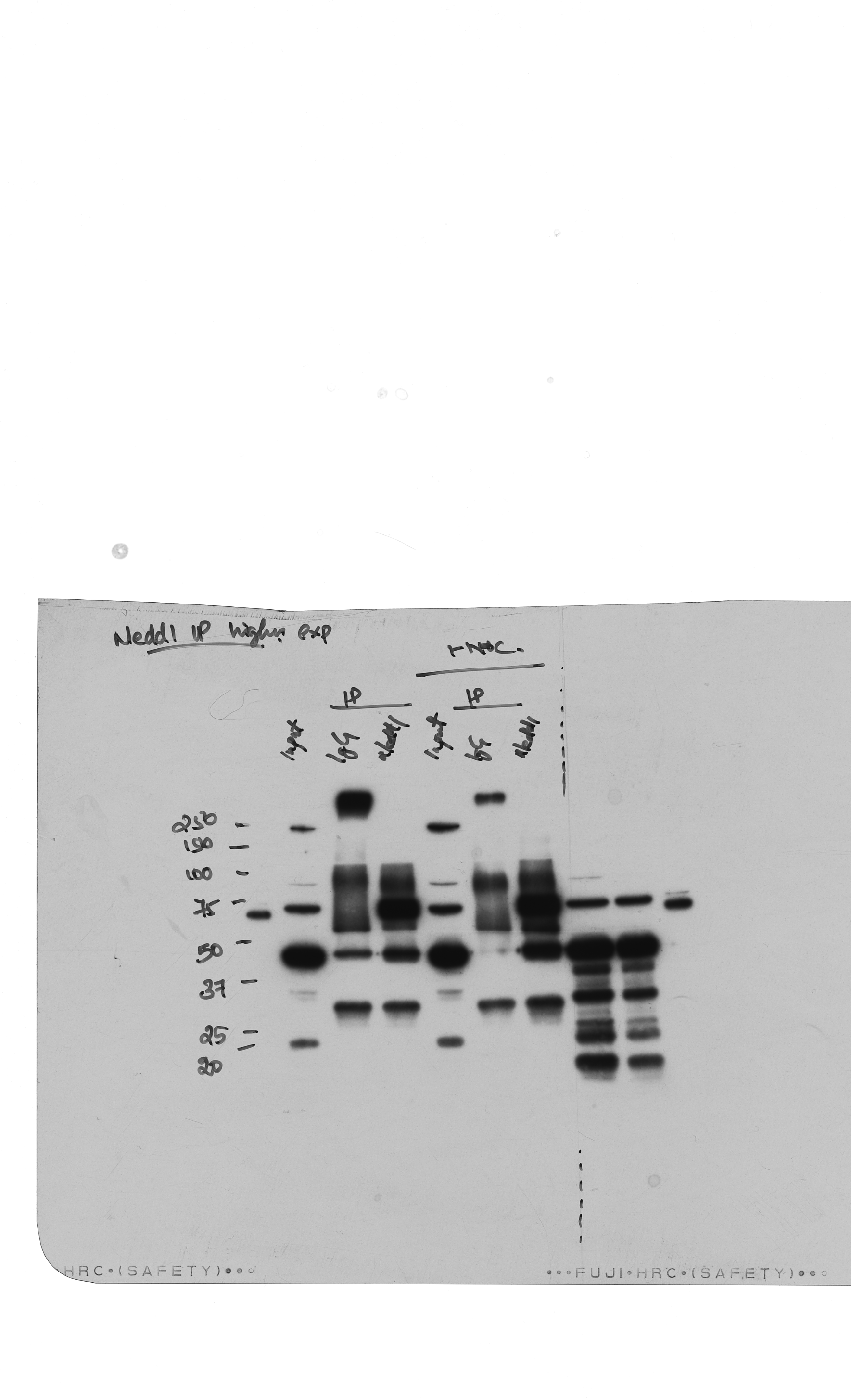

Supplement: Supplementary file 4 — Source Data [file 41467_2023_35955_MOESM4_ESM.zip › Source data_2ndrev_JL/Uncropped Western scans/Supplementary Figure 3E/Supplementary Fig 3E_NEDD1 IP/Supplimentary Fig SF 3E_nedd1 ip exposure 1.tif]

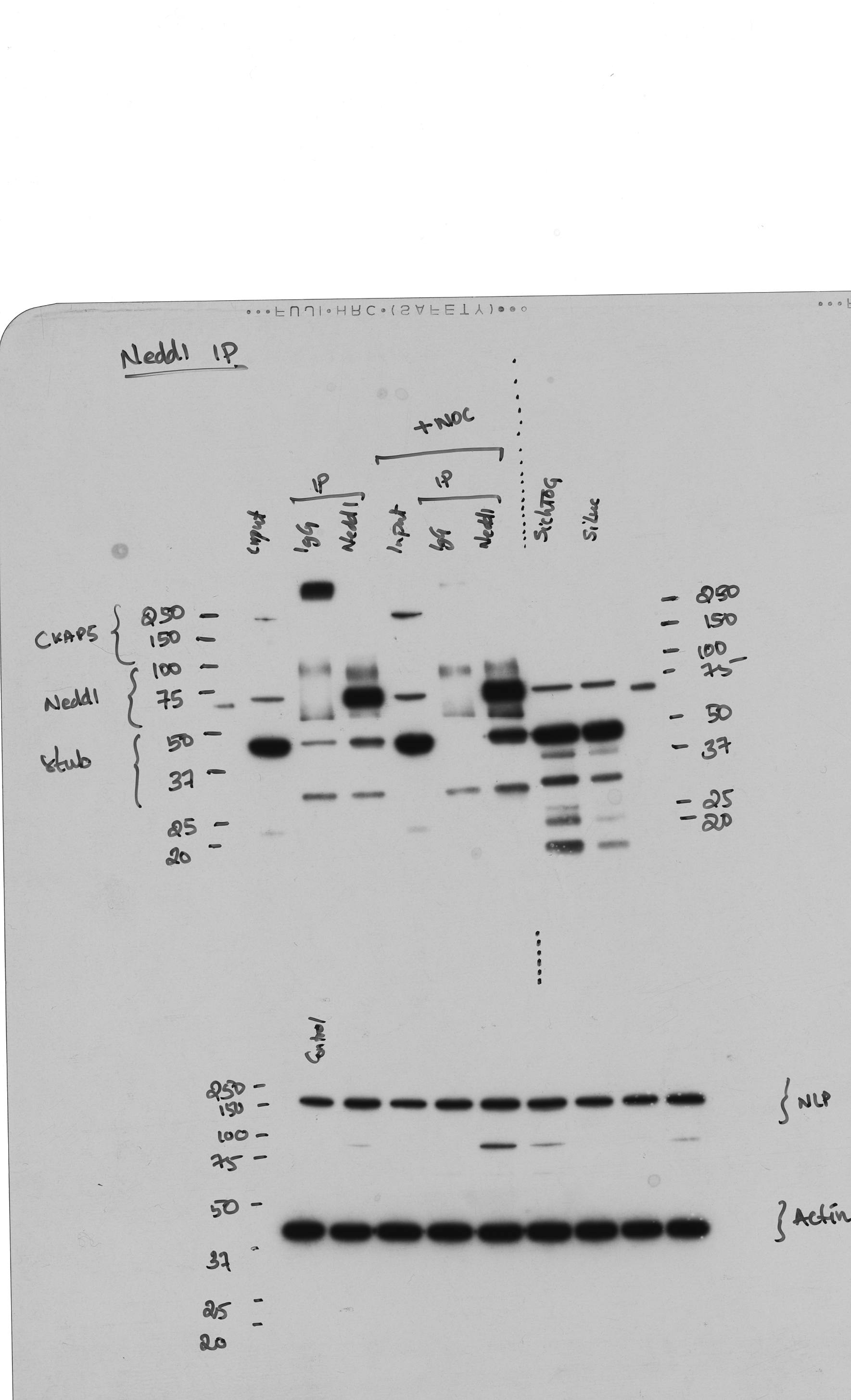

Supplement: Supplementary file 4 — Source Data [file 41467_2023_35955_MOESM4_ESM.zip › Source data_2ndrev_JL/Uncropped Western scans/Supplementary Figure 3E/Supplementary Fig 3E_NEDD1 IP/Supplimentary Fig SF 3E_nedd1 ip exposure 2.tif]

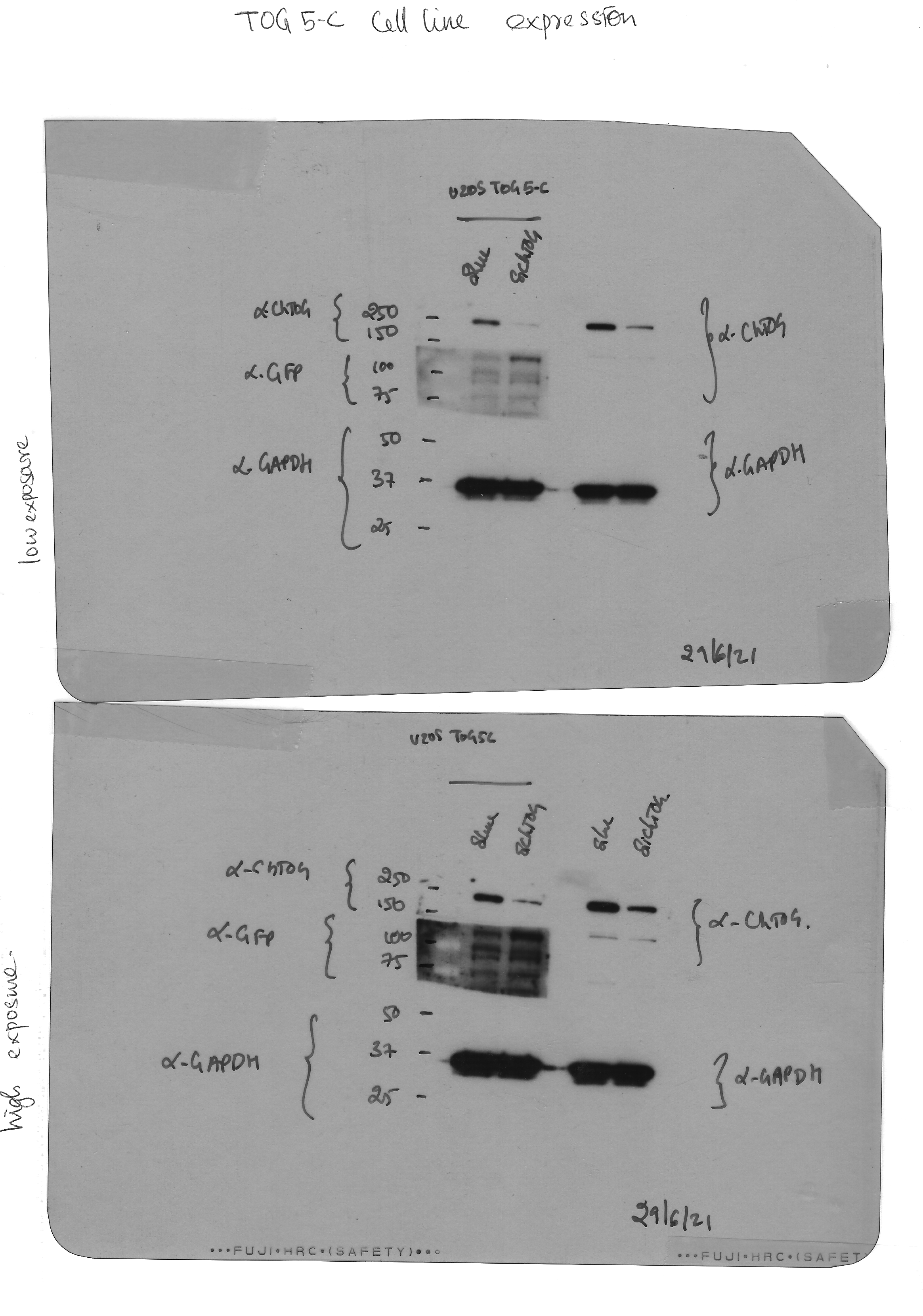

Supplement: Supplementary file 4 — Source Data [file 41467_2023_35955_MOESM4_ESM.zip › Source data_2ndrev_JL/Uncropped Western scans/Supplementary Figure 4B/Supplementary Fig 4B_CKAP5 KD in CKAP5-5C construct/Supplimentary Fig SF 4B_CKAP5 KD in CKAP5-5C construct.tiff]
